# Protein deglycase DJ-1 deficiency aggravates acute viral myocarditis by promoting apoptosis via reducing Dusp1 expression

**DOI:** 10.1038/s41419-025-08185-9

**Published:** 2025-11-28

**Authors:** Pengcheng Yan, Shuai Feng, Baona Li, Wenchao Yin, Leiyan Wang, Gang Zhao, Mengqi Guo, Zhaoyang Wang, Haitao Yuan

**Affiliations:** 1 Department of Cardiology, Shandong Provincial Hospital Affiliated to Shandong First Medical University, Jinan, China; 2 Department of Cardiology, Shandong Provincial Hospital, Shandong University, Jinan, China; 3 Jinan Key Laboratory of Cardiovascular Diseases, Jinan, China; 4 Department of Cardiology, The Affiliated Hospital of Qingdao University, Qingdao, China

**Keywords:** Apoptosis, Viral infection

## Abstract

Myocardial apoptosis is a cardinal process in acute viral myocarditis (VMC) instigated by coxsackievirus B3 (CVB3) infection. The anti-apoptotic regulator protein deglycase DJ-1 (DJ-1) is implicated in several pathological processes, yet its role in CVB3-induced VMC remains obscure. In this study, we decipher the protective role of DJ-1 against VMC. We found decreased DJ-1 expression in CVB3-infected H9C2 cells and VMC mouse heart tissues. DJ-1 knockout exacerbated VMC severity and apoptosis. Conversely, recovery of DJ-1 levels showed protective effects in vitro and in vivo. We observed downregulation of Dusp1 in DJ-1-deficient mice hearts and H9C2 cells with DJ-1 silencing. Subsequent in vitro assays corroborated that downregulation of DJ-1 amplifies apoptosis in H9C2 cells following CVB3 infection. This effect is mediated through the suppression of Dusp1 expression, thereby triggering the activation of the P38MAPK signaling pathway. Overall, our findings underscore DJ-1 as an appealing therapeutic target for VMC, with its anti-apoptotic effects in CVB3-induced VMC mediated in part via the Dusp1/P38MAPK signaling pathway. This novel understanding of DJ-1’s regulation might provide fresh insights into the pathogenesis of VMC and a potential therapeutic avenue for VMC.

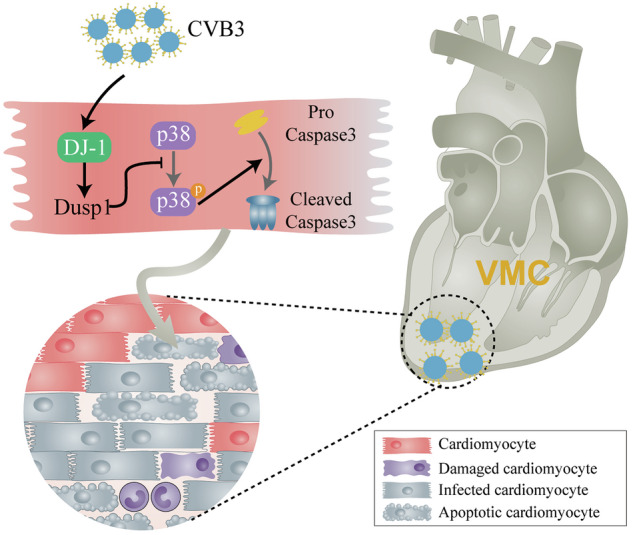

## Introduction

Myocarditis, characterized by inflammation and injury of the heart muscle, predominantly affects infants and adolescents. It can be caused by various pathogens, such as viruses, bacteria, fungi, Chlamydia, rickettsia, protozoa, and toxins, with group B Coxsackievirus (CVB3) being the most common [1–3]. Notably, recent research has found a connection between severe acute respiratory syndrome coronavirus 2 (the causative agent of Coronavirus disease-19) and myocarditis [4, 5]. The incidence of viral myocarditis (VMC) has significantly increased in recent years [6]. Although spontaneous recovery is common, approximately 20% of cases advance to a chronic stage, culminating in dilated cardiomyopathy and congestive heart failure [7, 8]. The pathogenesis of VMC involves direct or indirect myocardial damage mediated by viral infection and host immune response [9]. Cardiomyocyte injury results in the death of cardiomyocytes, which then triggers the release of pro-inflammatory cytokines. This amplifies the inflammatory response, leading to deterioration in cardiac function and ultimately heart failure. Given the challenges in studying the pathogenesis of VMC in humans, researchers have primarily relied on animal models, particularly mice. Despite extensive research over the years, our understanding of the underlying mechanism of VMC is still incomplete, and there are currently no effective therapies available [10].

Although the mechanism of CVB3-induced VMC is not fully understood, apoptosis has been reported to play a vital role. In 1994, Kawano et al. reported multifocal cardiomyocyte apoptosis in patients with chronic myocarditis through myocardial biopsy for the first time [11]. Subsequently, Henke et al. and Kyto et al. observed a significant number of apoptotic cardiomyocytes in mice infected with CVB3, suggesting the involvement of cardiomyocyte apoptosis in the pathogenesis of myocarditis [12, 13]. Apoptosis is a distinct form of cell death characterized by specific morphological features. The internucleosomal fragmentation of genomic DNA is a biochemical hallmark of apoptosis. Caspases, which are cysteine proteases, play a role in executing the morphological changes and DNA fragmentation during apoptosis. CVB3 expresses two viral proteases, 2A and 3C, both of which can induce apoptosis by activating the exogenous Caspase-8-mediated pathway and the endogenous mitochondria-mediated apoptosis pathway. Recent studies have demonstrated that inhibiting cell apoptosis could be a potential treatment for VMC [14–18].

DJ-1 is a 23 kDa protein containing 189 amino acids, initially identified as an oncogene. It plays crucial roles in transcription regulation, mitochondrial function, proteolysis, and autophagy [19]. Given its anti-oxidant and anti-apoptosis effects, DJ-1 has been extensively studied in cardiovascular disease, demonstrating protective effects against ischemia-reperfusion injury, pulmonary hypertension, cardiac hypertrophy, and heart failure [20–22]. DJ-1 activates several pro-survival and proliferative pathways, including nuclear factor erythroid 2-related factor, Akt, and extracellular signal-regulated protein kinases 1 and 2 pathways [23–25]. However, the role of DJ-1 in VMC and its influence on apoptosis in VMC remain nebulous. This study aims to elucidate the impact of DJ-1 on apoptosis induced by CVB3 and shed light on the potential underlying mechanisms.

## Materials and methods

### Animals and viruses

All C57BL/6 mice were male and aged 6–8 weeks. WT mice were supplied by the Experimental Animal Center of Shandong University. DJ-1-deficient mice were kindly provided by Professor Mingxiang Zhang (Qilu Hospital, Cheeloo College of Medicine, Shandong University, Jinan, Shandong), and their genotypes were determined using the following primers: 5′-GATAGCTTTCCGGGACACAC-3′; 5′-TCCATCAGCTCCTCCACCTCT-3′; 5′-TAAGTTGGGTAACGCCAGGGT-3′. All animals were housed in the Experimental Animal Center of Shandong Provincial Hospital Affiliated to Shandong First Medical University (Shandong, P.R. China). All animal experiments and programs have been approved by the Animal Care and Use Committee of Shandong Provincial Hospital. HeLa cells were used to prepare the CVB3 and measure the virus titers by TCID_50_ as described before [26, 27].

### Animal experiments

The mice in VMC groups were injected with 10^5^ TCID_50_ of CVB3 intraperitoneally at day 0, while the mice in control groups were injected with PBS (*n* = 12/group). To examine the role of DJ-1 in vivo, mice were injected intravenously with adeno-associated virus encoding DJ-1 (AAV-DJ-1) or adeno-associated virus vector (AAV-NC) 14 days before CVB3 infection. The AAV-DJ-1 and AAV-NC were purchased from Vigene Biosciences. Recording the survival and body weight of mice every day. Take an echocardiography of all the mice at day 7. Then, all mice were sacrificed, and the heart tissues and serum were harvested for further analysis.

### Cell culture and treatment protocol

The H9C2 cell line was cultured in DMEM (Gibco, USA), supplemented with 1% penicillin-streptomycin and 10% fetal bovine serum (FBS) (Gibco, USA) at 37 °C in an incubator with 5% CO_2_. The Lipo8000™ RNAi MAX transfection reagent (Beyotime) was used for the transient transfection of small interfering (si) RNA into H9C2 according to the protocol recommended by the manufacturer. After being transfected with siRNA targeting DJ-1 (si-DJ-1) or a control scrambled sequence(si-NC) for 24 h, H9C2 cells were infected with CVB3(MOI = 10) to stimulate H9C2 apoptosis. After another 24 h of stimulation, the levels of apoptosis were examined. To further identify whether DJ-1 can ameliorate apoptosis, the Adenovirus encoding DJ-1 (Adv-DJ-1) or Adenovirus vector (Adv-GFP) was used to infect the H9C2 cells 24 h before CVB3 infection. Next, to identify whether DJ-1 deficiency affects Dusp1, Lentivirus encoding Dusp1 (LV-Dusp1) or Lentivirus vector (LV-NC) was added 48 h before CVB3. LV-Dusp1 and LV-NC were synthesized by GenePharma (Shanghai, China).

### Mouse cardiac echocardiography

All mice were anesthetized with 2% isoflurane. Echocardiographic measurements were conducted using an echocardiography system (Vevo 2100, Canada). Left ventricular ejection fraction (LVEF) and left ventricular fractional shortening (LVFS) were calculated in M-mode.

### Serological index measurement

The concentration of cardiac troponin I (cTNI), IL-1β, IL-6, and TNF-α in the serum was detected using Ean LISA Kit (Research Cloud Biology) according to the manufacturer’s guidelines.

### Histopathological analysis immunohistochemistry

Inflammatory infiltration and myocardial lesions were assessed by histopathology. After fixing in 4% paraformaldehyde, the heart samples were embedded in paraffin and sectioned (5 μm thick). Hematoxylin and eosin staining were used following the manufacturing guidelines (Servicebio, Wuhan, China). The degree of inflammation and score for severity were determined as follows: level 0 = no inflammation, level 1: < 25% inflammation, level 2: 25–50% inflammation, level 3: 50–75% inflammation, and level 4: more than 75% inflammation or with necrosis. Two trained pathologists performed the analysis independently.

### Immunofluorescence

Heart sections were blocked with 5% normal donkey serum (Dako) for 1 h at room temperature. Then, the sections were incubated with primary antibodies at 4 °C overnight. Subsequently, the second antibody was applied at 37 °C for 1 h. And then co-stained the nucleus with DAPI. Sections were observed and images were collected by Fluorescent Microscopy (Zeiss, Germany).

### TUNEL staining

Cellular apoptosis following the CVB3 infection was detected by the TUNEL assay. Paraffin sections of the hearts were stained by the Fluorescein (FITC) TUNEL Cell Apoptosis Assay Kit (Beyotime, C1088). Cells were counterstained with DAPI to detect the nucleus and examined by fluorescence microscopy.

### Flow cytometry

PE Annexin V Apoptosis Detection Kit (BD Biosciences, USA) was used to detect the apoptosis rate of H9C2 cells. Briefly, H9C2 cells were collected using 0.25% Trypsin without EDTA (Gibco). Centrifugation for 1000r, 5 min, and washed with PBS twice, then resuspended the cells in 100 μl 1× binding buffer. To achieve double staining, 5 μl of 7-Amino-Actinomycin (7-AAD) and PE Annexin-V were added to each tube and the mixture was incubated at room temperature in the dark for 15 min. Subsequently, adding 400 μL of 1× binding buffer to each tube and analyzing the cells within 15 min by FACS Calibur flow cytometry (BD LSRFortessa, USA).

### Quantitative real-time PCR

Total RNA was extracted from heart tissues or H9C2 cells using TRIzol reagent (Accurate Biology, China) following the manufacturer’s guidelines. One microgram of RNA from each sample was used to reverse transcribe in a reaction buffer containing MMLV-RT and oligo (dT) primers (Accurate Biology, China). PCR amplification was performed using SYBR green real-time PCR kits (Accurate Biology, China) as we previously described [27]. The sequences of primers used in qPCR are as follows:

**Table:** 

Gene	Species	Forward primer	Reverse primer
Gapdh	Rat	5′–TCTCTGCTCCTCCCTGTTCT–3′	5′–ATCCGTTCACACCGACCTTC–3′
DJ-1	Rat	5′–AGTCGGCTTTGGTGAAGGAG–3′	5′–GCCAATGGGTGCGATGTAAC–3′
CVB3		5′–TGGAACGCAAGTATTCGGCT–3′	5′–ATGGCCGAACCACAGAACAT–3′
Dusp1	Rat	5′–GGGCACCTCTACTACAACGG–3′	5′–TGTGATGGGGCTTTGAAGGT–3′
Dusp5	Rat	5′–ACCACCCACCTACACTACAAG–3′	5′–GCGGAACTGCTTGGTCTTCAT–3′
Socs3	Rat	5′–CCCCGCTTTGACTGTGTACT–3′	5′–GTACCAGCGGGATCTTCTCG–3′
Tnfaip3	Rat	5′–AACGGTGATGGAAACTGCCT –3′	5′–AGTGTCGTAGCAAAGTCCCG–3′
Gbp3	Rat	5′–AATTCCCTGAAGCCCAAGCA –3′	5′–AACTCATCATCACGCAGGCT –3′
Ptgs2	Rat	5′–GATGACGAGCGACTGTTCCA –3′	5′–TGGTAACCGCTCAGGTGTTG–3′
Isg15	Rat	5′–GAGCAAGTCTCCCAAGACCA–3′	5′–GTTAGGCCATACTCCCCCAG –3′
Gapdh	Mice	5′-TGTCTCCTGCGACTTCAACA–3′	5′–GGTGGTCCAGGGTTTCTTACT–3′
DJ-1	Mice	5′–CGAGCCGGGATCAAAGTCAC–3′	5′–ACCACCACATCGTATGGTCC–3′
Dusp1	Mice	5′–TTGTCGCTCCTTCTTCGCTT–3′	5′–TCAGCGTTGGGCACGATATG–3′

The relative expression levels were analyzed using the 2^−ΔΔCt^ method.

### Western blot analysis

Myocardial tissue and cells were fully lysed by RIPA lysate (Solarbio, China). The concentration was determined by the bicinchoninic acid (BCA) protein assay (Beyotime, P0010). The extracted proteins were heated at 100 °C for 10 min. Western blot assay was performed as we described previously. The following antibodies were used: DJ-1 (Abcam, ab76008), VP1 (GeneTex, GTX132346), Pro-caspase 3 (Cell Signaling Technology, #9662), Cleaved caspase 3(Cell Signaling Technology, #9664), Dusp1 (Santa, sc-373841), P38MAPK (Cell Signaling Technology, #8690), pP38MAPK (Cell Signaling Technology, #4511), Gapdh polyclonal antibody (Proteintech, No. 10494-1-AP).

### Statistical analysis

Statistical analyses were performed using SPSS version 20.0 and GraphPad Prism version 7. The results were presented as the mean ± SD. Log-rank test was used to analyze the survival rate of mice. Statistical significance between two groups was determined by Student's *t*-test. For comparisons of multiple groups, one-way ANOVA by post-test (Tukey) analysis was used. *P* < 0.05 is considered a statistically significant difference.

### Ethics approval

This study was performed in line with the principles of the Declaration of Helsinki. Approval was granted by the Ethics Committee of Shandong Provincial Hospital (Date 2020/SDU.SPHEARC-A099).

## Results

### DJ-1 expression was reduced after CVB3 infection

**Fig. 1:**
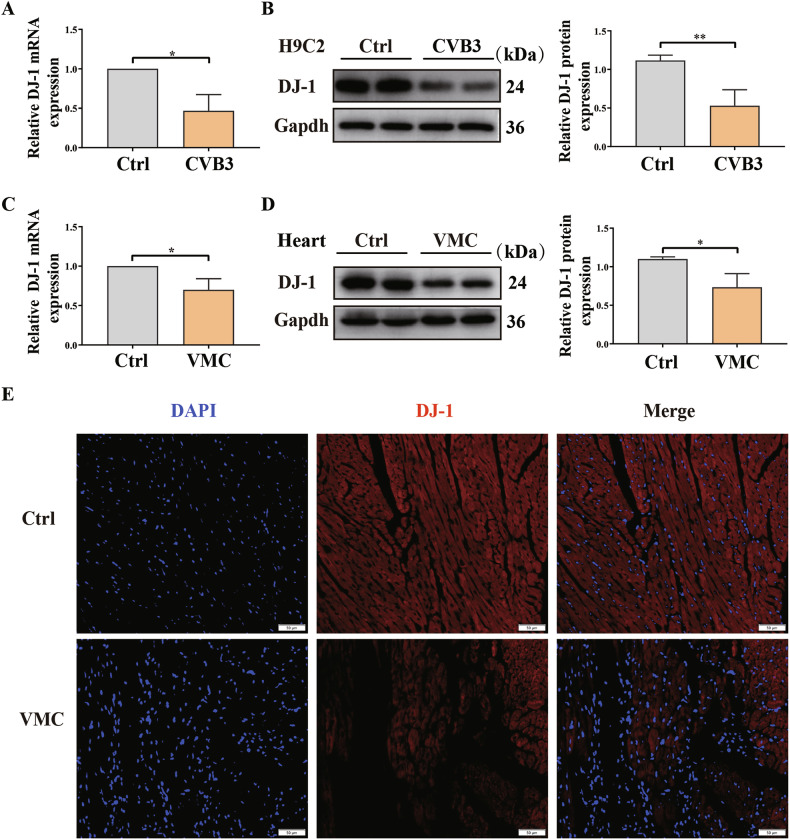
Expression of DJ-1 was reduced after CVB3 infection. **A** Quantitative qRT-PCR analysis of DJ-1 mRNA levels in the control and CVB3-infected H9C2 cells. **B** Western blot analysis of DJ-1 protein expression in H9C2 cells. Ctrl negative control (MEM), CVB3 CVB3 infected groups. **C** Quantitative qRT-PCR analysis of DJ-1 mRNA levels in the heart of Control and VMC mice (*n* = 3). **D** Western blot analysis of DJ-1 protein expression in the heart of Control and VMC mice (*n* = 3). **E** Immunofluorescence staining of DJ-1 in the heart of control and VMC mice. Nuclei were counterstained with DAPI (blue); red staining indicates DJ-1 exists (scale bar:50 μm, *n* = 6). (^*^P < 0.05; ^**^P < 0.01).

To elucidate the potential changes in DJ-1 expression following CVB3 infection, we assessed DJ-1 levels in both CVB3-infected H9C2 cells and mouse heart tissues. In our in vitro experiments, we utilized H9C2 cells infected with CVB3 to assess changes in DJ-1 expression. Through RT-qPCR and western blot analyses, we observed a notable reduction in DJ-1 levels in the cells exposed to the virus (Fig. 1A, B). Similarly, in our in vivo study using mouse heart tissues, we found a consistent pattern of decreased DJ-1 expression following CVB3 infection. This was proved by both RT-qPCR and western blot techniques (Fig. 1C, D). Immunofluorescence assays corroborated these trends (Fig. 1E). These results suggest that CVB3 infection reduced the level of DJ-1, implying a potential role for DJ-1 in the progression of VMC.

### DJ-1 deletion intensifies CVB3-induced myocardium injury

**Fig. 2:**
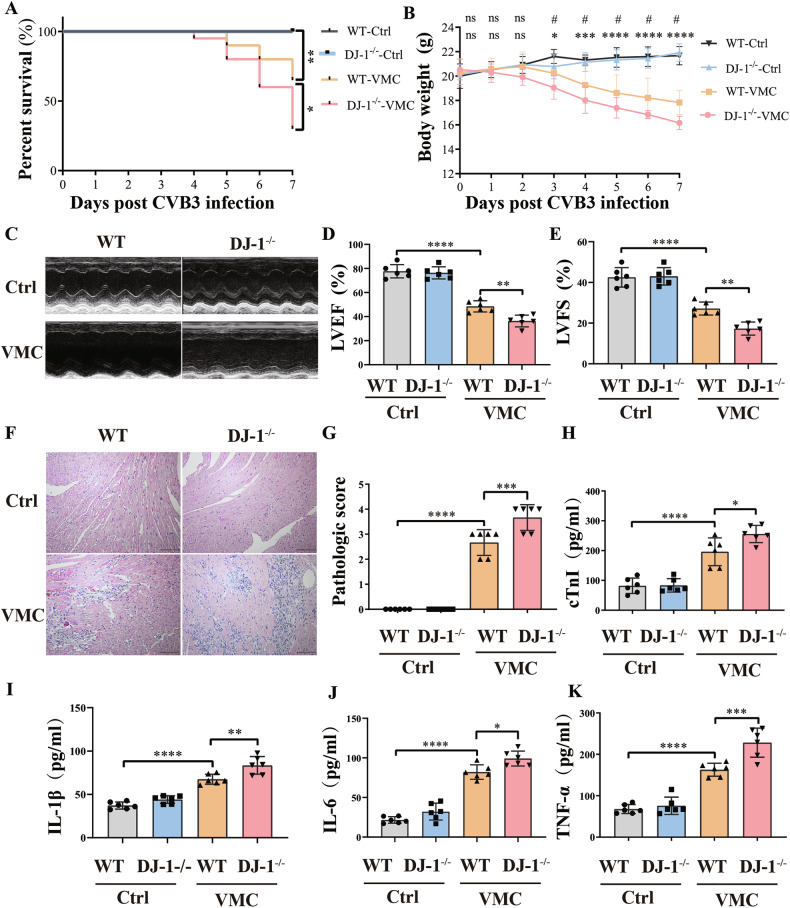
DJ-1 deletion aggravates CVB3-induced myocardium injury. **A** Survival curve of the mouse in different groups (*n* = 20). Survival proportions at day 7 were 100% for the wild-type control group and the DJ-1 deletion control group. **B** Body weight change of mice from day 0 to day 7. **C**–**E** Representative transthoracic M-mode echocardiogram was performed on day 7. LVEF and LVFS were measured (*n* = 6). **F** Hematoxylin–eosin staining to observe the inflammatory response to myocarditis. The red-stained area shows myocardial tissue, and blue staining shows inflammatory cell infiltration (scale 100 µm, *n* = 6). **G** The severity of myocarditis was scored using a standard 0–4 grading scale (*n* = 6). **H** Serum myocardial injury markers, cardiac troponin I (cTnI) were measured by ELISA (*n* = 6). **I**–**K** Serum inflammation markers IL-1β, IL-6, and TNF-α were measured by ELISA (*n* = 6). Data are shown as mean ± SD (WT-Ctrl, normal wild type mice; DJ-1^−/−^-Ctrl, normal DJ-1 deletion mice; WT-VMC, CVB3-infected wild type mice; DJ-1^−/−^-VMC, CVB3-infected DJ-1 deletion mice. ^*^WT-VMC vs WT-Ctrl; ^#^DJ-1^−/−^-VMC vs WT-VMC; one-way ANOVA by post-test (Tukey) analysis was used. ns nonsignificant, ^*^P < 0.05, ^**^P < 0.01, ^***^P < 0.001, ^****^P < 0.0001, ^#^*P* < 0.05).

To investigate the effects of DJ-1 on VMC, we utilized DJ-1 knockout mice (DJ-1^−/−^) in our VMC animal model. The survival analysis revealed a lower survival rate in the DJ-1^−/−^ group compared to the wild-type VMC group (Fig. 2A). Furthermore, the DJ-1^−/−^ group exhibited a greater loss in body weight compared to the wild-type VMC group (Fig. 2B). We next sought to decipher the potential role of DJ-1 in cardiac function. DJ-1 deletion exacerbated the impairment of cardiac contractility and diastolic function caused by CVB3 infection (Fig. 2C–E). Hematoxylin and eosin staining demonstrated that DJ-1 knockout mice infected with CVB3 had a marked increase in inflammatory infiltration (Fig. 2F, G). We also examined the concentrations of cardiac troponin I and pro-inflammatory cytokines, such as IL-1β, IL-6, and TNF-α, in peripheral blood samples. Our data revealed an elevation in these biomarkers, indicating myocardial injury and inflammation in the wild-type VMC group. Notably, these levels were further augmented in the DJ-1^−/−^ group, suggesting an exacerbating role of DJ-1 deficiency in VMC severity (Fig. 2H–K). These results collectively suggest thatthe absence of DJ-1 exacerbates the progression of CVB3-induced VMC in vivo.

### DJ-1 deficiency aggravates myocardial apoptosis without a change in viral replication

**Fig. 3:**
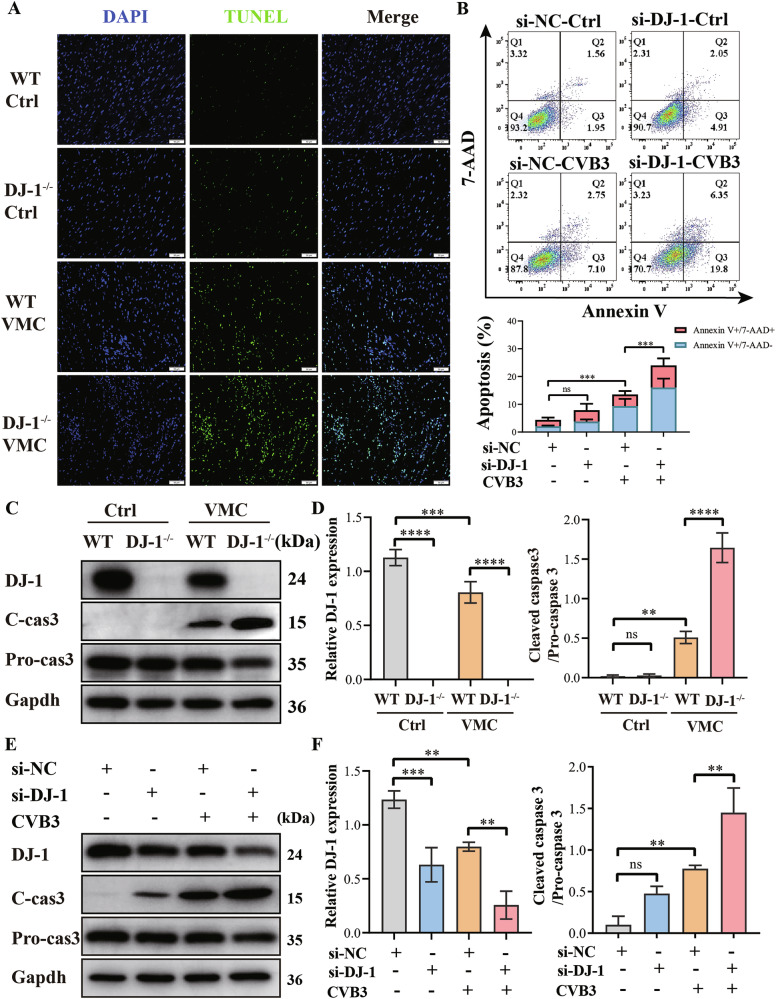
DJ-1 deficiency increases myocardial apoptosis. **A** TUNEL assays were used to detect apoptosis in the heart of mice. Green staining indicates apoptotic cells; Nuclei were counterstained with DAPI (blue). WT-Ctrl, normal wild type mice, DJ-1^−^-Ctrl, normal DJ-1 deletion mice, WT-VMC, CVB3-infected wild type mice, DJ-1^−/−^-VMC, CVB3-infected DJ-1 deletion mice. Scale bar:50 µm, *n* = 6). **B** H9C2 cells transfected with siRNA targeting DJ-1 (si-DJ-1) or a control scrambled sequence (si-NC) were infected with CVB3. Apoptosis was determined by annexin V-PE/7AAD staining assay. **C**, **D** The expression levels of DJ-1, Pro-caspase-3, and Cleaved caspase-3 in the heart were assessed by western blotting. **E**, **F** The expression levels of DJ-1, Pro-caspase-3, and Cleaved caspase-3 in H9C2 cells were assessed by western blotting. The data are represented as the mean ± SEM. *n* = 3. One-way ANOVA by post-test (Tukey) analysis was used. ns nonsignificant, ^*^P < 0.05; ^**^P < 0.01; ^***^P < 0.001; ^****^P < 0.0001).

To elucidate the mechanism by which DJ-1 protects against VMC, we first examined viral replication. RT-qPCR and Western blot analyses revealed that DJ-1 deficiency did not alter viral replication (Fig S1A–C). Given the established association between apoptosis and VMC (Fig S2A–D), and the known anti-apoptotic effects of DJ-1 (Fig S2E–H), we next turned our attention to the anti-apoptotic role of DJ-1. We observed an increased number of apoptotic cells (TUNEL-positive) in the VMC groups relative to control groups, an effect that was intensified by DJ-1 deletion (Fig. 3A). Flow cytometry revealed an increase of apoptotic cells in CVB3-infected H9C2 cells, with this effect being greater in si-DJ-1 groups (Fig. 3B). The levels of cleaved caspase3 were elevated, and pro-caspase3 levels were decreased, thereby the caspase3 activity was elevated in the VMC group compared to the control group, which indicated an increase of apoptosis, with these effects being more pronounced in the DJ-1 deletion groups (Fig. 3C, D). Meanwhile, the protein levels of cleaved caspase3 in H9C2 cells were increased, and pro-caspase3 were decreased in the CVB3 group than the Control group, and the caspase3 activity was elevated in the CVB3 group than the Control group, which was more significant in the si-DJ-1 groups (Fig. 3E, F). Overall, these findings suggest that DJ-1 deficiency accelerates myocardial apoptosis induced by CVB3 infection.

### DJ-1 overexpression inhibits apoptosis induced by CVB3 infection and relieves myocarditis

**Fig. 4:**
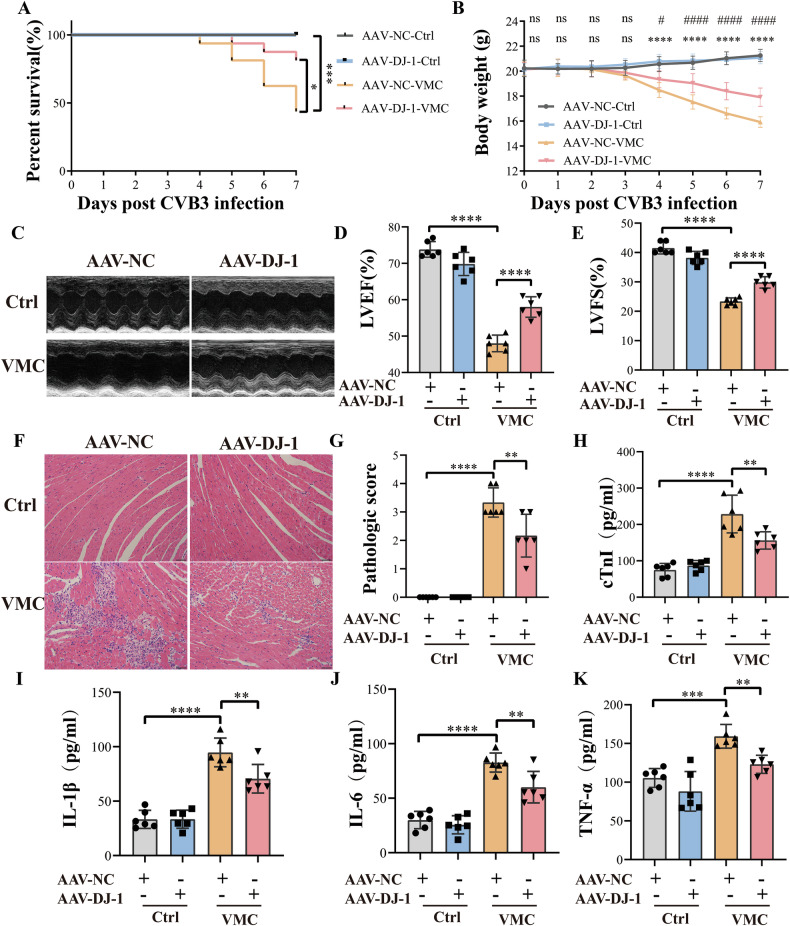
DJ-1 overexpression relieved VMC in mice. **A** Survival curve of the mouse in different groups (*n* = 20). Survival proportions at day 7 were 100% for the control group and the DJ-1 overexpression control group. **B** Body weight change of mice from day 3 to day 7. **C**–**E** Representative transthoracic M-mode echocardiogram was performed on day 7. LVEF and LVFS were measured (*n* = 6). **F** Hematoxylin–eosin staining to observe the inflammatory response to myocarditis. The red-stained area shows myocardial tissue, and blue staining shows inflammatory cell infiltration (scale bar: 100 µm, *n* = 6). **G** The severity of myocarditis was scored using a standard 0–4 grading scale (*n* = 6). **H** Serum myocardial injury markers, cardiac troponin I (cTnI), were measured by ELISA (*n* = 6). **I**–**K** Serum inflammation markers IL-1β, IL-6, and TNF-α were measured by ELISA (*n* = 6). Data are shown as mean ± SD. (AAV-NC, wild type mice injected with vector adeno-associated virus; AAV-DJ-1, wild type mice injected with adeno-associated virus encoding DJ-1. ^*^AAV-NC-VMC vs AAV-NC-Ctrl; ^#^AAV-DJ-1-VMC vs AAV-NC-VMC; one-way ANOVA by post-test (Tukey) analysis was used. ns nonsignificant, ^*^P < 0.05, ^**^P < 0.01, ^***^P < 0.001, ^****^P < 0.0001, ^#^*P* < 0.05, ^###^*P* < 0.001, ^####^*P* < 0.0001).

**Fig. 5:**
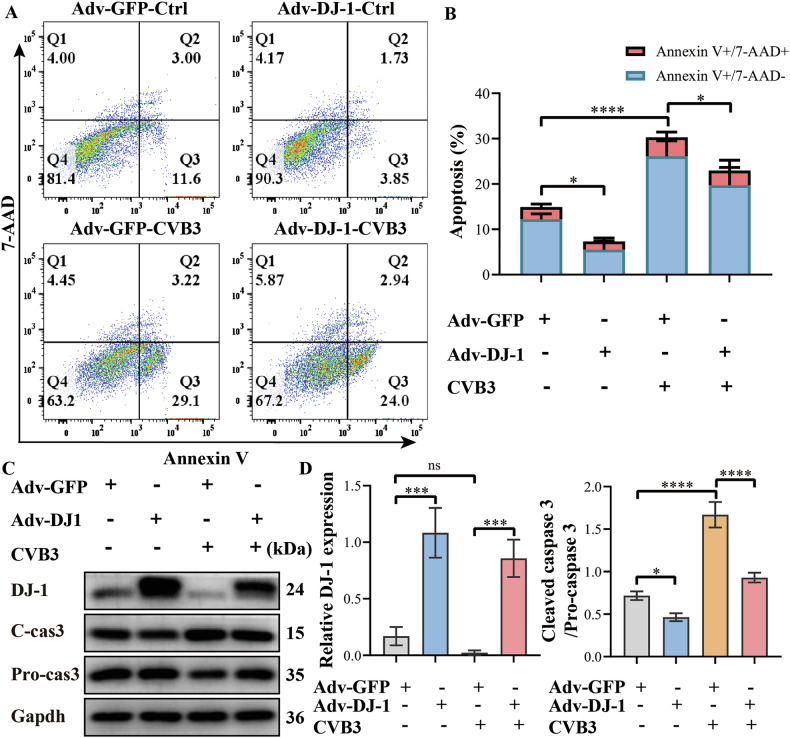
DJ-1 overexpression inhibited apoptosis induced by CVB3 infection. **A**, **B** Flow cytometry was used to detect the apoptosis rate. **C**, **D** The expression levels of DJ-1, Pro-caspase-3, and Cleaved caspase-3 were assessed by western blotting. (Adv-GFP, H9C2 cells transfected with vector adenovirus; Adv-DJ-1, H9C2 cells transfected with adenovirus encoding DJ-1. The data are represented as the mean ± SEM. *n* = 3. One-way ANOVA by post-test (Tukey) analysis was used. ns nonsignificant, ^*^P < 0.05; ^**^P < 0.01; ^***^P < 0.001; ^****^P < 0.0001).

To further identify whether DJ-1 can ameliorate VMC, WT mice were injected with AAV-DJ-1 to restore the level of DJ-1. Results showed that DJ-1 overexpression improved the survival rate of myocarditis, mitigated the loss in body weight (Fig. 4A, B). Besides, DJ-1 overexpression alleviated the impairment of cardiac contractility and diastolic function caused by CVB3 infection, manifested as increases in EF and FS (Fig. 4C–E). Hematoxylin and eosin staining demonstrated that DJ-1 overexpression decreases the inflammatory infiltration in the heart of VMC mice (Fig. 4F, G). Furthermore, the levels of cardiac troponin I and pro-inflammatory cytokines (IL-1β, IL-6, and TNF-α) in peripheral blood were decreased in the DJ-1 overexpression group than in the control VMC group (Fig. 4H–K). Flow cytometry revealed a decrease in apoptotic cells in the DJ-1 overexpression group than the control group (Fig. 5A, B). Besides, DJ-1 overexpression exhibited decreased protein levels of cleaved caspase-3 and increased protein levels of pro-caspase-3, thereby reducing the caspase-3 activity, which indicated a decrease in apoptosis (Fig. 5C, D). All the above findings suggest that DJ-1 overexpression alleviates myocardial apoptosis induced by CVB3 infection and relieves myocarditis.

### DJ-1 deficiency downregulates Dusp1 gene expression

**Fig. 6:**
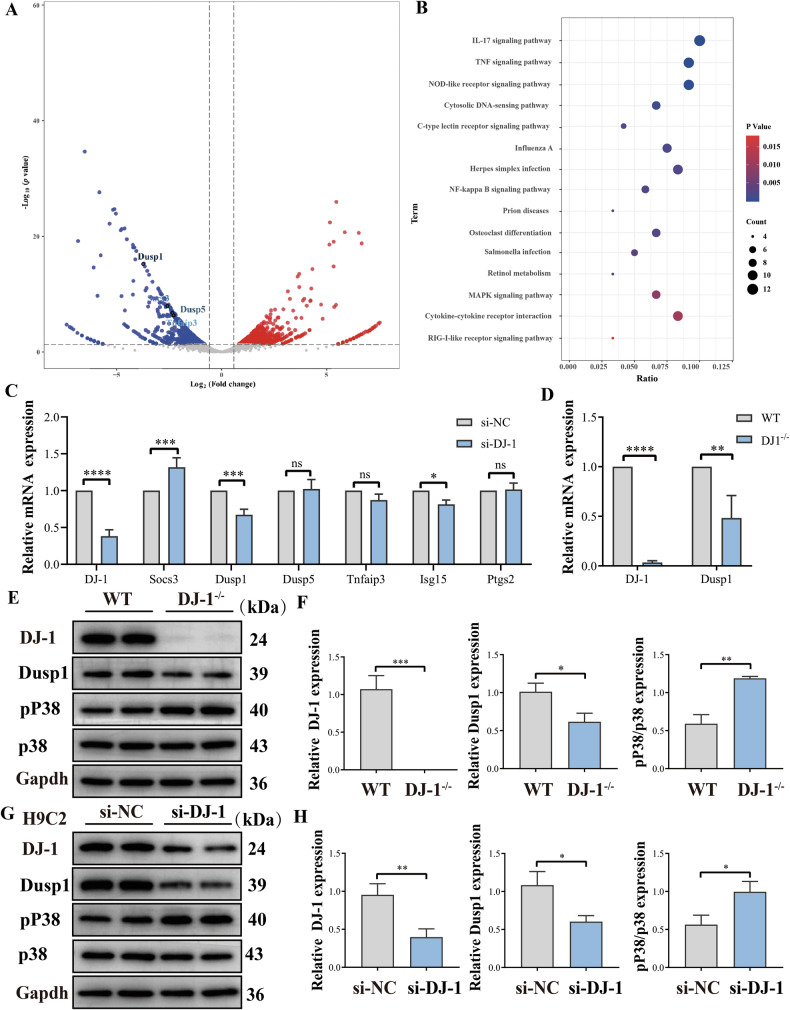
DJ-1 deficiency downregulates Dusp1 gene expression. **A** RNA transcriptome sequencing of the heart from DJ-1^−/−^ and WT mice. The fold change of all genes with a significant difference was presented in the volcano plot. **B** KEGG analysis of downregulated genes in DJ-1^−/−^ mice compared with the WT type. **C** H9C2 cells were transfected with siRNA targeting DJ-1 (si-DJ-1) or a control scrambled sequence (si-NC). The mRNA levels of DJ-1 and Dusp1 were determined by RT-qPCR at the indicated time. **D** Quantitative RT-qPCR analysis of Dusp1 mRNA level in the heart of DJ-1 deletion mice and wild-type mice. **E**–**H** Depressed protein level of Dusp1 and increased activation of the P38MAPK pathway in the DJ-1 deficiency groups compared with the control (the data are represented as the mean ± SEM. One-way ANOVA by post-test (Tukey) analysis was used. *n* = 3. ^*^P < 0.05; ^**^P < 0.01; ^***^P < 0.001; ^****^P < 0.0001).

Previous studies have established that inhibiting cell apoptosis serves as a potential treatment strategy for VMC [14–18]. Consistent with this, our research also demonstrates that DJ-1 exhibits anti-apoptotic and protective effects in CVB3 infection. To decipher the mechanism by which DJ-1 regulates apoptosis, RNA transcriptome sequencing was performed on the hearts of 6-week-old DJ-1^−/−^ mice and wild-type mice. In the volcano plot, the downregulated genes occupied a dominant position with a total of 191 after DJ-1 knockout, in which the expression of Dusp1, Dusp5, Socs3 and Tnfaip3 was significantly reduced (Fig. 6A). Correspondingly, the KEGG analysis of downregulated genes in the DJ-1^−/−^ group compared with the WT group indicated that they were enriched in pathways including MAPK and NF-κB signaling pathway (Fig. 6B). Prior research has highlighted a close relationship between apoptosis and several genes related to MAPK and NF-κB signaling pathway, including Dusp1, Dusp5, Socs3, and Tnfaip3 [28–31]. Among these, Dusp1 was the most significantly impacted in our results (Fig. 6C). PCR results confirmed this change in vivo (Fig. 6D). Given that Dusp1 is a dual-specificity phosphatase that specifically targets and inactivates P38, we posited that DJ-1’s regulatory effect on apoptosis may rely on Dusp1. To validate this hypothesis, we examined Dusp1, P38, and pP38 expression via western blotting. Our results indicated that Dusp1 was downregulated in conditions of DJ-1 deficiency both in vivo and in vitro, leading to activation of the P38MAPK pathway (Fig. 6E–H).

### Dusp1 mediates the anti-apoptosis effect of DJ-1 on CVB3 infection

**Fig. 7:**
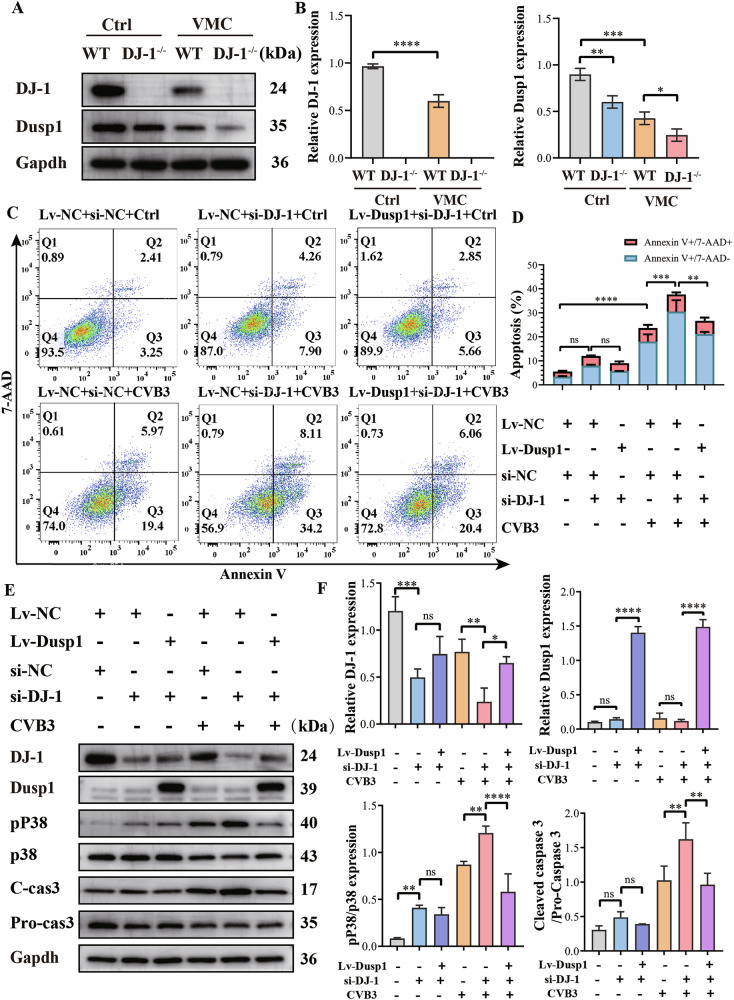
Dusp1 mediates the anti-apoptosis effect of DJ-1 on CVB3 infection. **A**, **B** Western blot analyses revealed that DJ-1 deficiency downregulated Dusp1 upon CVB3 infection in the heart of mice. The expression levels of DJ-1 and Dusp1 in hearts from DJ-1^−/−^ and WT mice were assessed by western blotting. **C**, **D** Flow cytometry was used to detect the apoptosis rate. **E**, **F** The expression levels of DJ-1, Dusp1, P38 MAPK, pP38 MAPK, Pro-caspase-3, and Cleaved caspase-3 were assessed by western blotting. Lv-Dusp1, H9C2 cells transfected with lentivirus encoding Dusp1. Lv-NC, H9C2 cells transfected with the lentivirus vector. si-DJ-1, H9C2 cells transfected with siRNA targeting DJ-1. si-NC, H9C2 cells transfected with a control scrambled sequence. The data are represented as the mean ± SEM. *n* = 3. One-way ANOVA by post-test (Tukey) analysis was used. ^*^P < 0.05; ^**^P < 0.01; ^***^P < 0.001; ^****^P < 0.0001).

We further investigated the expression of Dusp1 in the hearts of mice subjected to VMC by western blotting. Our findings revealed that under conditions of DJ-1 deficiency, the expression of Dusp1 was significantly downregulated (Fig. 7A, B). Subsequently, we explored the role of Dusp1 in apoptosis under conditions of DJ-1 deficiency with or without CVB3 infection. Overexpression of Dusp1 in H9C2 cells (via LV-Dusp1 transfection) mitigated apoptosis due to DJ-1 deficiency (Fig. 7C, D). Correspondingly, overexpression of Dusp1 in H9C2 cells reduced the elevated caspase-3 activity induced by CVB3 infection. Furthermore, Dusp1 overexpression significantly inhibited activation of the P38 MAPK pathway induced by CVB3 infection and DJ-1 deficiency (Fig. 7E, F). These findings confirm that Dusp1 mediates the anti-apoptotic effect of DJ-1 on CVB3 infection.

## Discussion

VMC, an inflammatory cardiac disease, has been a focus of intensive research with a particular emphasis on immunomodulatory therapies. Current literature underscores the pivotal effect of apoptosis in VMC. In this innovative study, we probed the function and mechanistic pathways of DJ-1 in VMC. Our findings reveal that DJ-1 deficiency not only exacerbates myocardial apoptosis, which intensifies the severity of VMC, but also amplifies inflammatory cell infiltration and the production of pro-inflammatory cytokines in VMC mice. Moreover, we have identified that DJ-1 overexpression and the gene overexpression of Dusp1 can inhibit apoptosis, subsequently ameliorating myocarditis due to the DJ-1 deficiency. To our knowledge, this is the first study confirming the potential therapeutic efficacy of DJ-1 in rescuing VMC and apoptosis via Dusp1.

Coxsackieviruses are encapsulated by infectious particles VP1–4 and a single-stranded RNA genome approximately 7400 nucleotides long, which functions as mRNA within the host cell. A growing body of evidence has reported that CVB3 infection can induce cardiomyocyte apoptosis [32, 33]. However, the precise mechanisms triggering this induction and the role of cardiomyocyte apoptosis in the subsequent pathogenesis of myocarditis remain elusive. Apoptosis can be triggered by a variety of stimuli, all of which involve the processing and activation of caspase 3 [34]. Caspase 3, an essential protein in the execution phase of cell death, exists in cells as a precursor protein. After infection of CVB3, Pro-caspase3 will cleave into cleaved caspase3 around 8 h [35]. This active form of caspase 3 can degrade specific substrates, leading to structural changes and loss of homeostatic regulation of cellular processes [36].

DJ-1, a protein with multifaceted roles, is most renowned for its anti-oxidant and anti-apoptotic effects [37]. Compared to wild-type mice, DJ-1-deficient mice appear more susceptible under conditions of cellular stress. For example, the myocardial infarction size of DJ-1-deficient mice was larger with more apoptotic cardiomyocytes, and a lot of studies have verified the protective effect of DJ-1 on ischemia/reperfusion injury [38]. Besides, mice lacking DJ-1 were more susceptible to pressure-overload left ventricular hypertrophy caused by total aortic constriction and angiotensin II [39]. Our previous studies demonstrated that DJ-1 has an effect on the phenotype of vascular smooth muscle cells, and the lack of DJ-1exacerbated the instability of atherosclerotic plaque [40]. Moreover, overexpression of DJ-1 protected cardiomyocytes from oxidative stress-induced cell death [41]. In the present study, our study is the first to report that DJ-1 expression is diminished after CVB3 infection, and DJ-1 deficiency exacerbates CVB3-induced myocardial injury and apoptosis. Nevertheless, DJ-1 overexpression inhibits CVB3-induced apoptosis and alleviates myocarditis.

The protective role of DJ-1 in preventing cell death has been well established, especially in I/R. Whether directly with ROS compounds (e.g., H_2_O_2_) or indirectly with ROS-induced stressors (e.g., hypoxia), deletion of DJ-1 resulted in increased susceptibility to oxidative stress-induced cell death in a wide range of cells, including MEFs, HeLa, COS7, Beas-2b, and cardiomyocytes [39, 42–48]. Overexpression or restoration of DJ-1 by adenoviral vectors can protect cells from death [49]. In addition to indirectly reducing cell death by scavenging ROS from cells, DJ-1 also prevents cell death by directly binding to mediators of the apoptotic pathway. DJ-1 prevents the activation of apoptosis signal-regulated kinase 1 (ASK1) through multiple pathways. Firstly, DJ-1 stabilizes the Trx1/ASK1 inhibitory complex and inhibits ASK1 release from Trx1 during oxidative stress [50]. Second, DJ-1 upregulates Trx1 expression through the Nrf2 pathway, thereby inhibiting ASK1 activation [51]. Further, under oxidative stress conditions, DJ-1 directly interacts with the nuclear death structural domain-associated protein (Daxx) and prevents Daxx translocation to the cytoplasm, thereby preventing Daxx from activating ASK1 and delaying apoptosis [52–54]. Besides, DJ-1 upregulates cell survival signaling pathways, including extracellular regulation of protein kinase 1/2 and protein kinase B [48, 55, 56]. Under oxidative stress, DJ-1 downregulates Bax expression and prevents cell death by inhibiting the transcriptional activity of the tumor suppressor p53 at the Bax promoter [57–59]. As a response to oxidative stress, DJ-1 activates p53, which then reduces DJ-1 through a negative feedback loop [60].

While DJ-1 is known to inhibit apoptosis, the specific mechanism by which DJ-1 regulates apoptosis in myocarditis remains unknown. Dual-specificity phosphatase-1(Dusp1, also called MKP-1), which is known to dephosphorylate and inactivate different mitogen-activated protein kinase (MAPK) isoforms, is highly expressed in the heart, lungs, and liver [61]. Besides, the expression of Dusp1 can be influenced by a variety of extracellular stimuli [62]. The role of Dusp1 in mediating apoptosis has been exhaustively studied in various models. However, the exact role of Dusp1 in VMC is unclear. In this study, Dusp1 expression was reduced in DJ-1-deficient mice and H9C2 cells, with simultaneous activation of the p38MAPK pathway. Furthermore, overexpression of Dusp1 was able to reverse the exacerbation of VMC and apoptosis due to DJ-1 deficiency. This suggests that DJ-1 may exert its anti-apoptotic effect through downregulating Dusp1, contributing to the alleviation of myocarditis.

Despite the groundbreaking findings, this study has certain limitations. While myocytes predominantly constitute the cardiac tissue, our study did not extend to evaluating the interactions between DJ-1 and other resident cell types like fibroblasts, macrophages, and T cells in the context of myocarditis. To substantiate our findings, future research employing myocardial-specific DJ-1 knockout mice is warranted. Moreover, the current study’s absence of clinical patient data necessitates further clinical investigations to corroborate the therapeutic potential of targeting DJ-1 in myocarditis.

In conclusion, this study provides the first evidence that DJ-1 deficiency exacerbates CVB3-induced acute VMC by promoting apoptosis by reducing Dusp1 expression, and DJ-1 overexpression could reverse this trend. Therefore, our findings suggest a promising therapeutic role of DJ-1 in treating VMC.

## Supplementary information

Reproducibility checklist

Supplemental Figure legends

Figure s1

Figure s2

Figure s3

Figure s4

Table s1 s2 s3.docx

Full and uncropped western blots
