# Supplementary material for: Protein deglycase DJ-1 deficiency aggravates acute viral myocarditis by promoting apoptosis via reducing Dusp1 expression
Source: Cell Death Dis. 2025 Nov 28;16(1):866. doi: 10.1038/s41419-025-08185-9 (PMC12663478; doi:10.1038/s41419-025-08185-9)
Supplement: Supplementary file 2 — Supplemental Figure legends [file 41419_2025_8185_MOESM2_ESM.docx]

**Fig s1 DJ-1 deficiency did not alter viral replication**

(A) RT-qPCR revealed DJ-1 deficiency did not alter viral replication. (B, C) Western blot analyses revealed DJ-1 deficiency did not alter viral replication. The data are represented as the mean ± SEM. n = 3. One-way ANOVA by post-test (Tukey) analysis was used. **p < 0.01; ***p < 0.001; ****p < 0.0001).

**Fig s2** **CVB3 infection induced apoptosis and DJ-1 protected against apoptosis**

(A, B, C, D) Western blot analyses revealed CVB3 infection induced apoptosis in the heart of mice and H9C2 cells. (E, F, G, H) Western blot analyses revealed the anti-apoptotic effects of DJ-1 in H9C2 cells. The data are represented as the mean ± SEM. n = 3. One-way ANOVA by post-test (Tukey) analysis was used. *p < 0.05; **p < 0.01; ****p < 0.0001).

**Fig**.s3 **DJ-1 overexpression relieved viral myocarditis in mice**

(A) Survival curve of the mouse in different groups(n=20). Survival proportions at day 7 were 100% for the control group and DJ-1 overexpression control group. (B) Body weight change of mice from day 3 to day 7. (C, D, E) Representative transthoracic M-mode echocardiogram was performed on day 7. LVEF and LVFS were measured(n=3). (F) Hematoxylin-eosin staining to observe the inflammatory response to myocarditis. The red stained area shows myocardial tissue, and blue staining shows inflammatory cell infiltration (Scale bar:100µm, n=6). (G)The severity of myocarditis was scored using a standard 0-4 grading scale(n=6). (H) Serum myocardial injury markers cardiac troponin I (cTnI) were measured by ELISA(n=6). (I, J, K) Serum inflammation markers IL-1β, IL-6, and TNF-α were measured by ELISA(n=6). Data are shown as mean ± SD. (Adv-GFP, wild type mice injected with vector adeno virus; Adv-DJ-1, wild type mice injected with adenovirus encoding DJ-1. *Adv-GFP-VMC vs Adv-DJ-1-Ctrl; #Adv-DJ-1-VMC vs Adv-GFP-VMC; One-way ANOVA by post-test (Tukey) analysis was used. ns, nonsignificant, *P<0.05, **P<0.01, ***P<0.001, ****P<0.0001, #P<0.05, ####P<0.0001).

**Fig s4 DJ-1 deficiency aggravated myocardial apoptosis**

(A) TUNEL assays and cTnT were used to detect the apoptosis in the heart of mice. Green staining indicates apoptotic cells; Myocyte were counterstained with cTnT (red). Nuclei were counterstained with DAPI (blue). (WT-Ctrl, normal wild type mice; DJ-1^-/-^-Ctrl, normal DJ-1 deletion mice; WT-VMC, CVB3-infected wild type mice; DJ-1^-/-^-VMC, CVB3-infected DJ-1 deletion mice. Scale bar:50µm, n=6. One-way ANOVA by post-test (Tukey) analysis was used).
