# Supplementary figures and images for: Protein deglycase DJ-1 deficiency aggravates acute viral myocarditis by promoting apoptosis via reducing Dusp1 expression
Source: Cell Death Dis. 2025 Nov 28;16(1):866. doi: 10.1038/s41419-025-08185-9 (PMC12663478; doi:10.1038/s41419-025-08185-9)

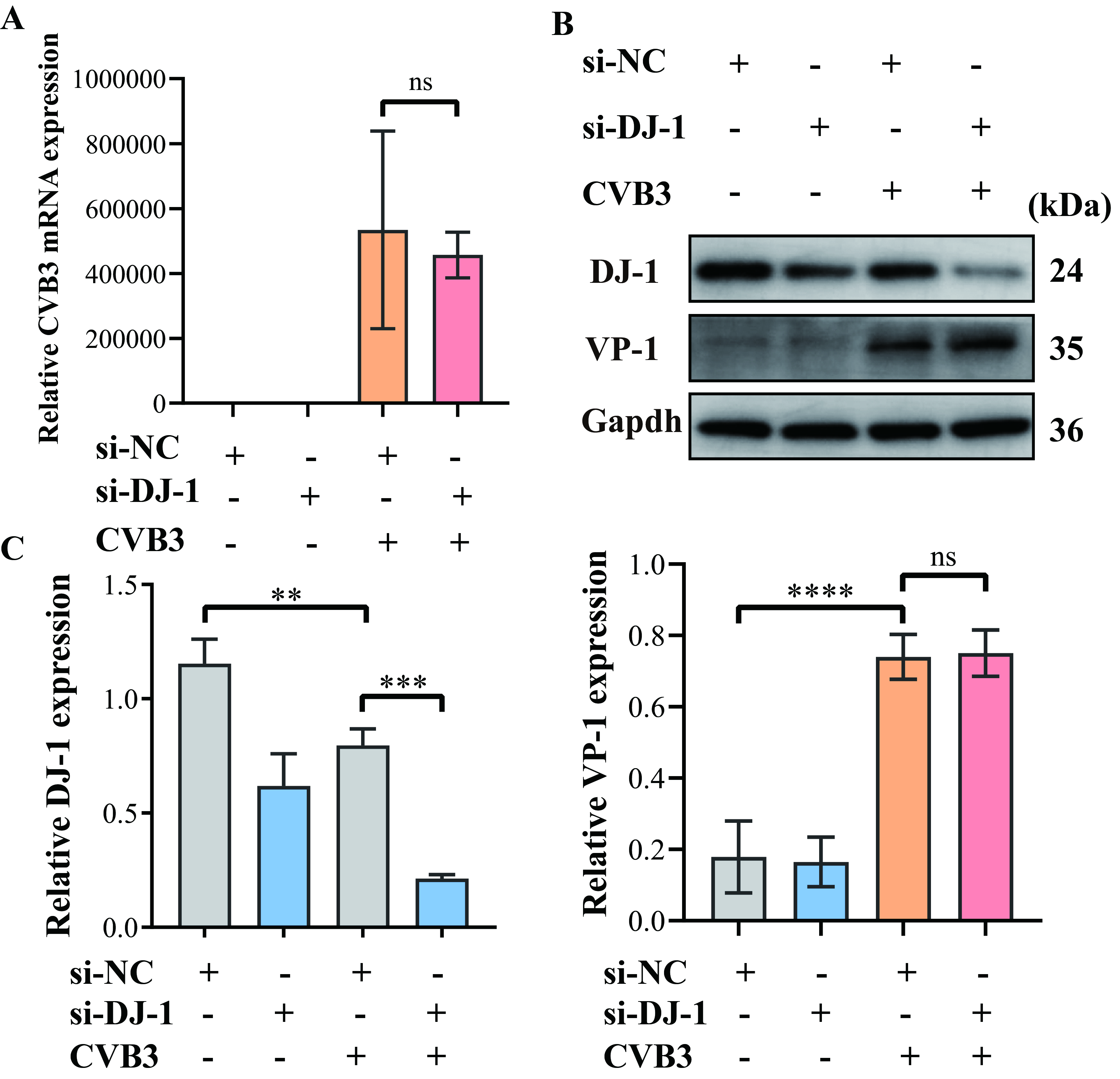

Supplement: Supplementary file 3 — Figure s1 [file 41419_2025_8185_MOESM3_ESM.tif]

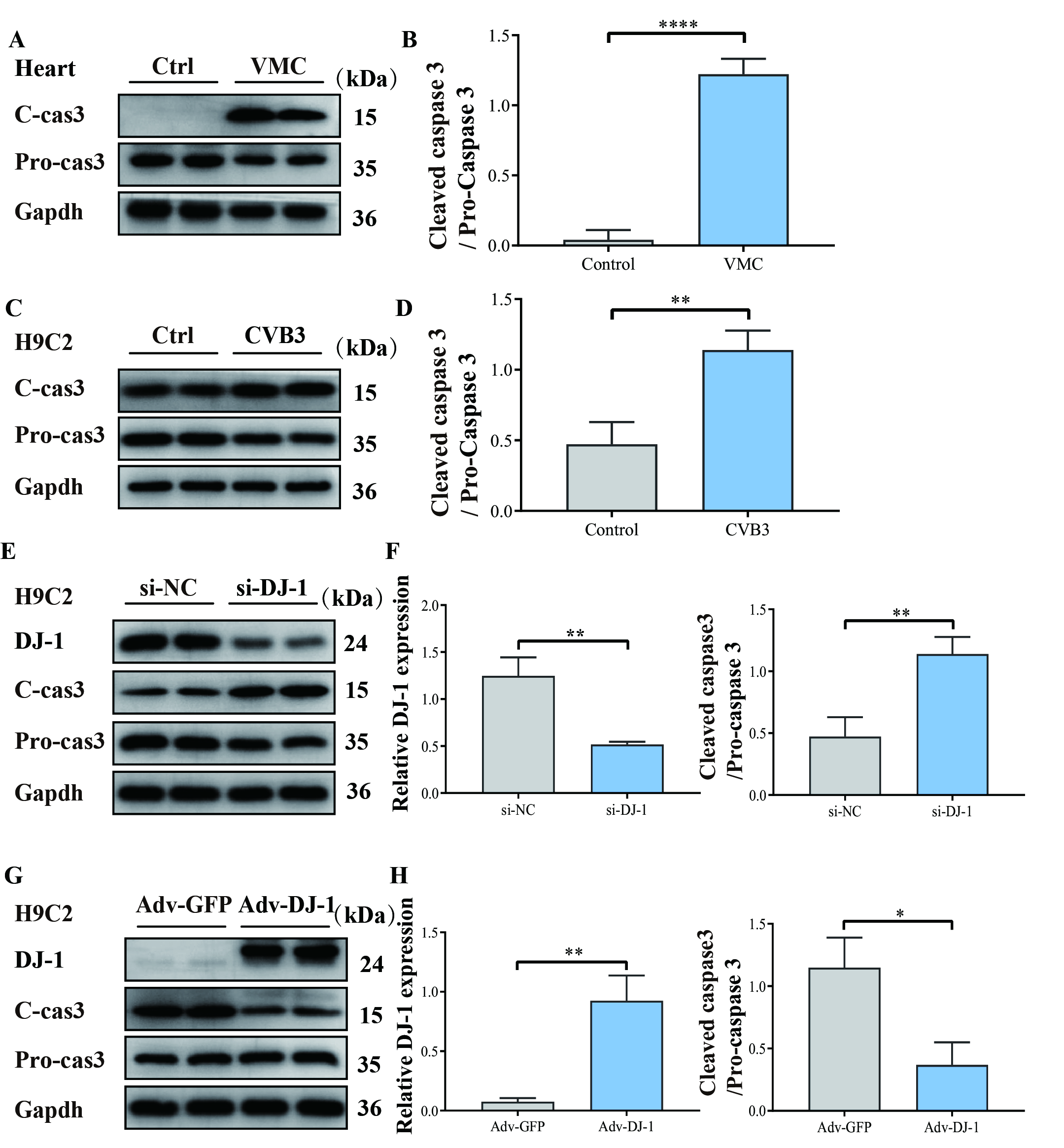

Supplement: Supplementary file 4 — Figure s2 [file 41419_2025_8185_MOESM4_ESM.tif]

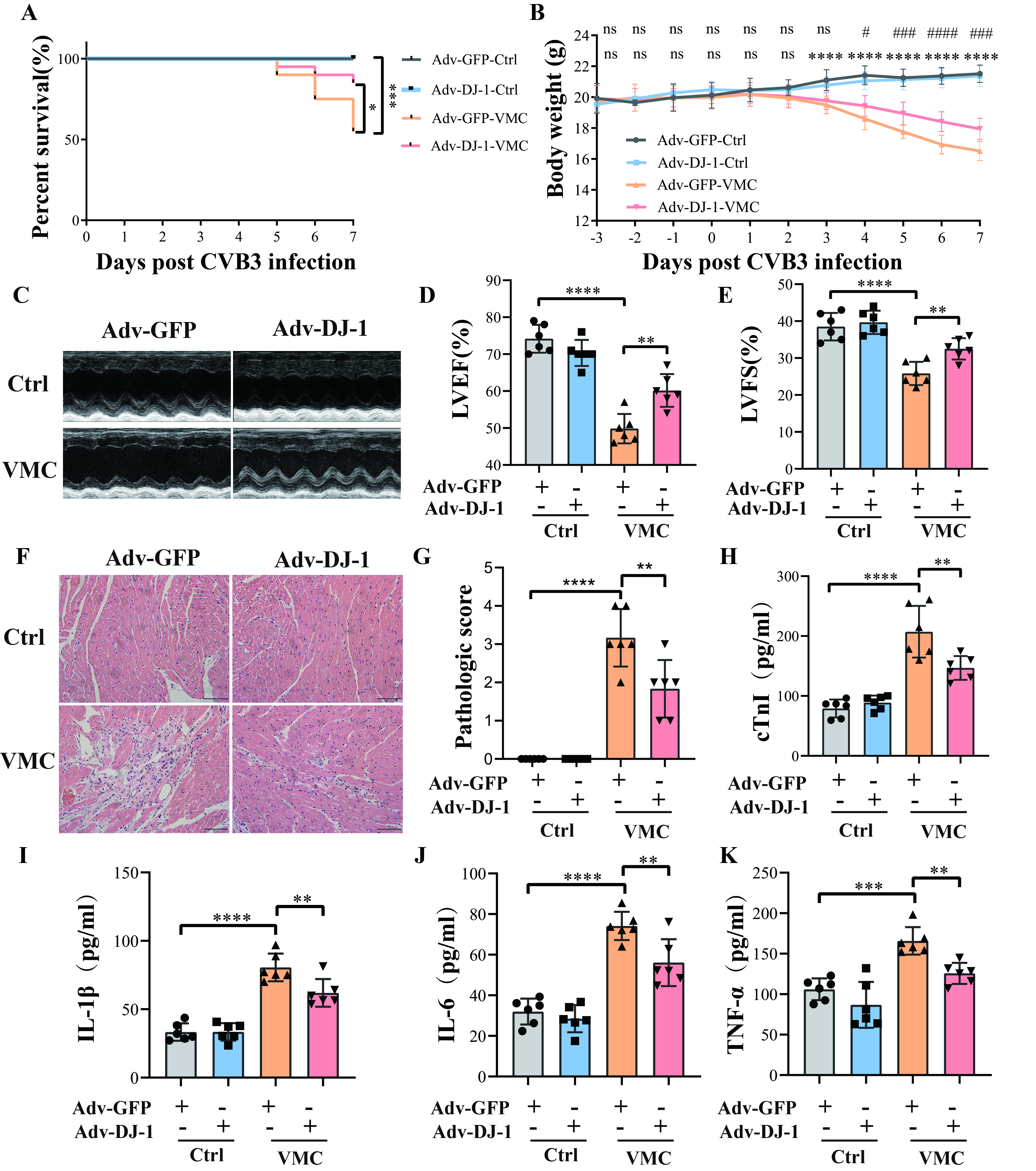

Supplement: Supplementary file 5 — Figure s3 [file 41419_2025_8185_MOESM5_ESM.tif]

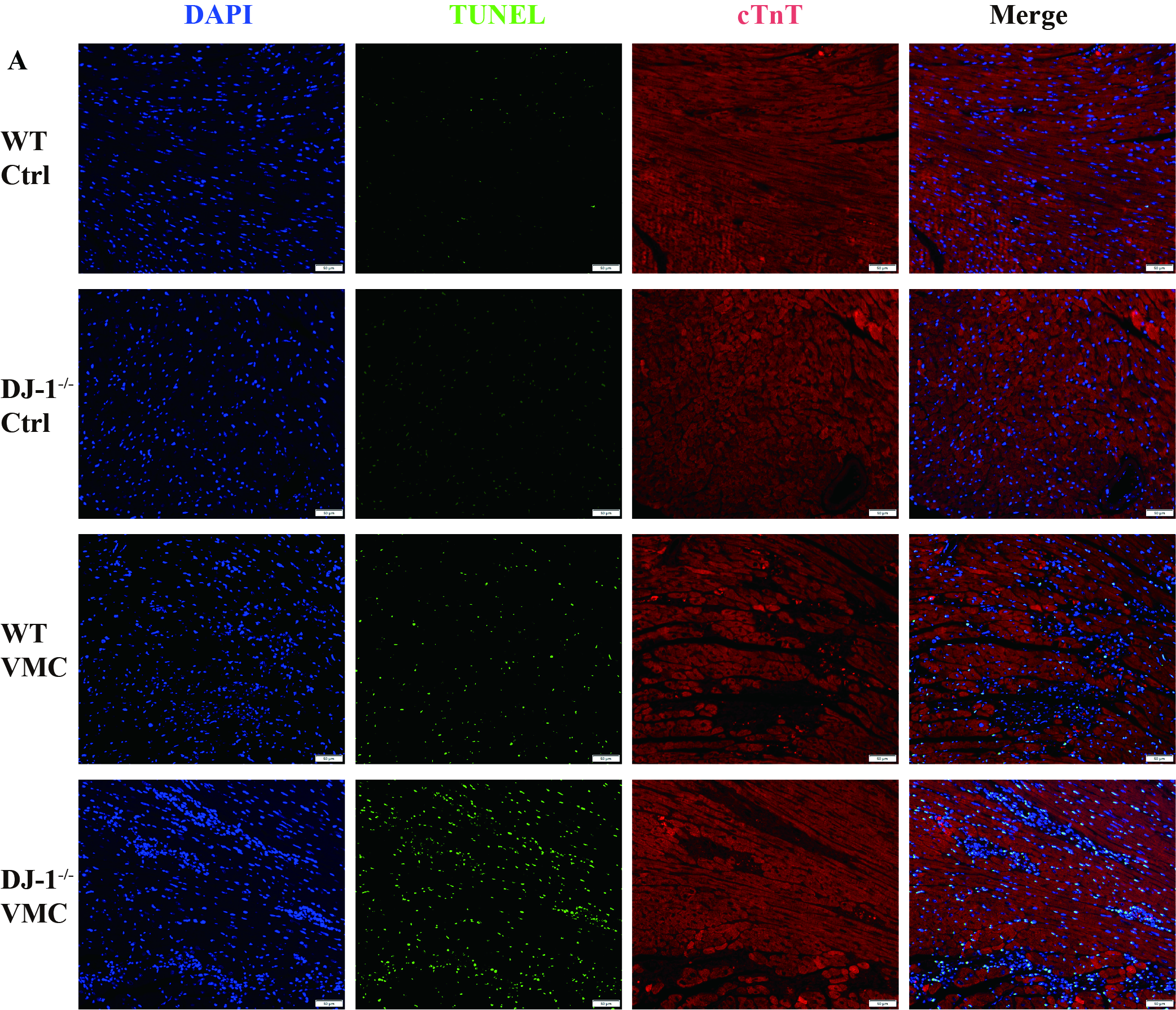

Supplement: Supplementary file 6 — Figure s4 [file 41419_2025_8185_MOESM6_ESM.tif]
