# Supplementary material for: Protein deglycase DJ-1 deficiency aggravates acute viral myocarditis by promoting apoptosis via reducing Dusp1 expression
Source: Cell Death Dis. 2025 Nov 28;16(1):866. doi: 10.1038/s41419-025-08185-9 (PMC12663478; doi:10.1038/s41419-025-08185-9)
Supplement: Supplementary file 7 — Table s1 s2 s3.docx [file 41419_2025_8185_MOESM7_ESM.docx]

**Table s1**

|  | WT-Ctrl | DJ-1^-/-^-Ctrl | WT-VMC | DJ-1^-/-^-VMC |
| --- | --- | --- | --- | --- |
| LVID-d(mm) | 3.30±0.19 | 3.32±0.14 | 3.40±0.08 | 3.47±0.05 |
| LVID-s(mm) | 1.80±0.11 | 1.85±0.11 | 2.59±0.05^****^ | 2.88±0.09^###^ |
| LVEF(%) | 77.51±5.25 | 76.25±5.08 | 48.51±4.38^****^ | 36.43±4.77^##^ |
| LVFS(%) | 45.33±5.49 | 44.11±5.06 | 23.81±2.67^****^ | 17.04±2.51^#^ |
| LVAW-d(mm) | 0.91±0.02 | 0.91±0.02 | 0.93±0.02 | 0.93±0.03 |
| LVAW-s(mm) | 1.25±0.02 | 1.24±0.02 | 1.09±0.03^****^ | 1.01±0.02^####^ |
| LVPW-d(mm) | 1.09±0.11 | 1.07±0.07 | 1.08±0.07 | 1.11±0.09 |
| LVPW-s(mm) | 1.55±0.05 | 1.54±0.01 | 1.44±0.05^**^ | 1.34±0.06^#^ |

LVID-d and LVID-s=left ventricular diastolic and systolic internal diameters; LVEF=left ventricular ejection fraction; LVFS=left ventricular short-axis shortening rate. LVAW-d and LVAW-s=left ventricular anterior wall thickness at end diastole and systole. LVPW-d and LVPW-s=left ventricular posterior wall thickness at end diastole and systole. Data are shown as mean ± SD. N=6. (WT-Ctrl, normal wild type mice; DJ-1^-/-^-Ctrl, normal DJ-1 deletion mice; WT-VMC, CVB3-infected wild type mice; DJ-1^-/-^-VMC, CVB3-infected DJ-1 deletion mice. *WT-VMC vs WT-Ctrl; # DJ-1^-/-^-VMC vs WT-VMC; One-way ANOVA by post-test (Tukey) analysis was used. ***p* < 0.01, *****p* < 0.0001, #*p* < 0.05, ##*p* < 0.01, ###*p* < 0.001, ####*p* < 0.0001).

**Table s2**

|  | AAV-NC-Ctrl | AAV-DJ-1-Ctrl | AAV-NC-VMC | AAV-DJ-1-VMC |
| --- | --- | --- | --- | --- |
| LVID-d(mm) | 3.13±0.09 | 3.10±0.06 | 3.24±0.04^*^ | 3.28±0.04 |
| LVID-s(mm) | 1.83±0.03 | 1.92±0.07 | 2.48±0.05^****^ | 2.30±0.04^####^ |
| LVEF(%) | 73.77±2.22 | 69.67±3.00 | 47.91±2.22^****^ | 58.19±2.76^####^ |
| LVFS(%) | 41.48±2.03 | 38.05±2.43 | 23.31±1.31^****^ | 29.81±1.86^####^ |
| LVAW-d(mm) | 0.92±0.03 | 0.91±0.03 | 0.93±0.02 | 0.93±0.03 |
| LVAW-s(mm) | 1.18±0.06 | 1.21±0.04 | 1.10±0.03^**^ | 1.02±0.02^#^ |
| LVPW-d(mm) | 1.07±0.12 | 1.06±0.08 | 1.07±0.10 | 1.10±0.07 |
| LVPW-s(mm) | 1.5±0.05 | 1.51±0.04 | 1.45±0.03 | 1.39±0.04 |

LVID-d and LVID-s=left ventricular diastolic and systolic internal diameters; LVEF=left ventricular ejection fraction; LVFS=left ventricular short-axis shortening rate. LVAW-d and LVAW-s=left ventricular anterior wall thickness at end diastole and systole. LVPW-d and LVPW-s=left ventricular posterior wall thickness at end diastole and systole. Data are shown as mean ± SD. N=6. (AAV-NC, wild type mice injected with vector adeno-associated virus; AAV-DJ-1, wild type mice injected with adeno-associated virus encoding DJ-1. *AAV-NC-VMC vs AAV-NC-Ctrl; #AAV-DJ-1-VMC vs AAV-NC-VMC; One-way ANOVA by post-test (Tukey) analysis was used. **p* < 0.05, ***p* < 0.01, *****p* < 0.0001, #*p* < 0.05, ####*p* < 0.0001).

**Table s3**

|  | Adv-GFP-Ctrl | Adv-DJ-1-Ctrl | Adv-GFP-VMC | Adv-DJ-1-VMC |
| --- | --- | --- | --- | --- |
| LVID-d(mm) | 3.16±0.12 | 3.15±0.06 | 3.44±0.12^***^ | 3.39±0.09 |
| LVID-s(mm) | 1.83±0.05 | 1.93±0.06 | 2.59±0.08^****^ | 2.33±0.09^####^ |
| LVEF(%) | 74.34±3.79 | 70.47±3.26 | 50.04±4.09^****^ | 60.21±4.53^##^ |
| LVFS(%) | 42.10±3.49 | 38.6±2.72 | 24.76±2.60^****^ | 31.32±3.2^##^ |
| LVAW-d(mm) | 0.90±0.03 | 0.91±0.04 | 0.92±0.03 | 0.91±0.04 |
| LVAW-s(mm) | 1.22±0.05 | 1.21±0.06 | 1.08±0.03^****^ | 1.16±0.12^####^ |
| LVPW-d(mm) | 1.11±0.10 | 1.10±0.09 | 1.1±0.06 | 1.11±0.06 |
| LVPW-s(mm) | 1.49±0.04 | 1.52±0.05 | 1.38±0.05^**^ | 1.46±0.06 |

LVID-d and LVID-s=left ventricular diastolic and systolic internal diameters; LVEF=left ventricular ejection fraction; LVFS=left ventricular short-axis shortening rate. LVAW-d and LVAW-s=left ventricular anterior wall thickness at end diastole and systole. LVPW-d and LVPW-s=left ventricular posterior wall thickness at end diastole and systole. Data are shown as mean ± SD. N=6. (Adv-GFP, wild type mice injected with vector adenovirus; Adv-DJ-1, wild type mice injected with adenovirus encoding DJ-1. *Adv-GFP-VMC vs Adv-GFP-Ctrl; #Adv-DJ-1-VMC vs Adv-GFP-VMC; One-way ANOVA by post-test (Tukey) analysis was used. ***p* < 0.01, ****p* < 0.001, *****p* < 0.0001, ##*p* < 0.01, ####*p* < 0.0001).
