# Supplementary material for: Protein deglycase DJ-1 deficiency aggravates acute viral myocarditis by promoting apoptosis via reducing Dusp1 expression
Source: Cell Death Dis. 2025 Nov 28;16(1):866. doi: 10.1038/s41419-025-08185-9 (PMC12663478; doi:10.1038/s41419-025-08185-9)

Fig 1B+s2D Repeat 1

Marker Ctrl Ctrl CVB3 CVB3

Gapdh   DJ-1   Cleaved caspase3   Pro-caspase3

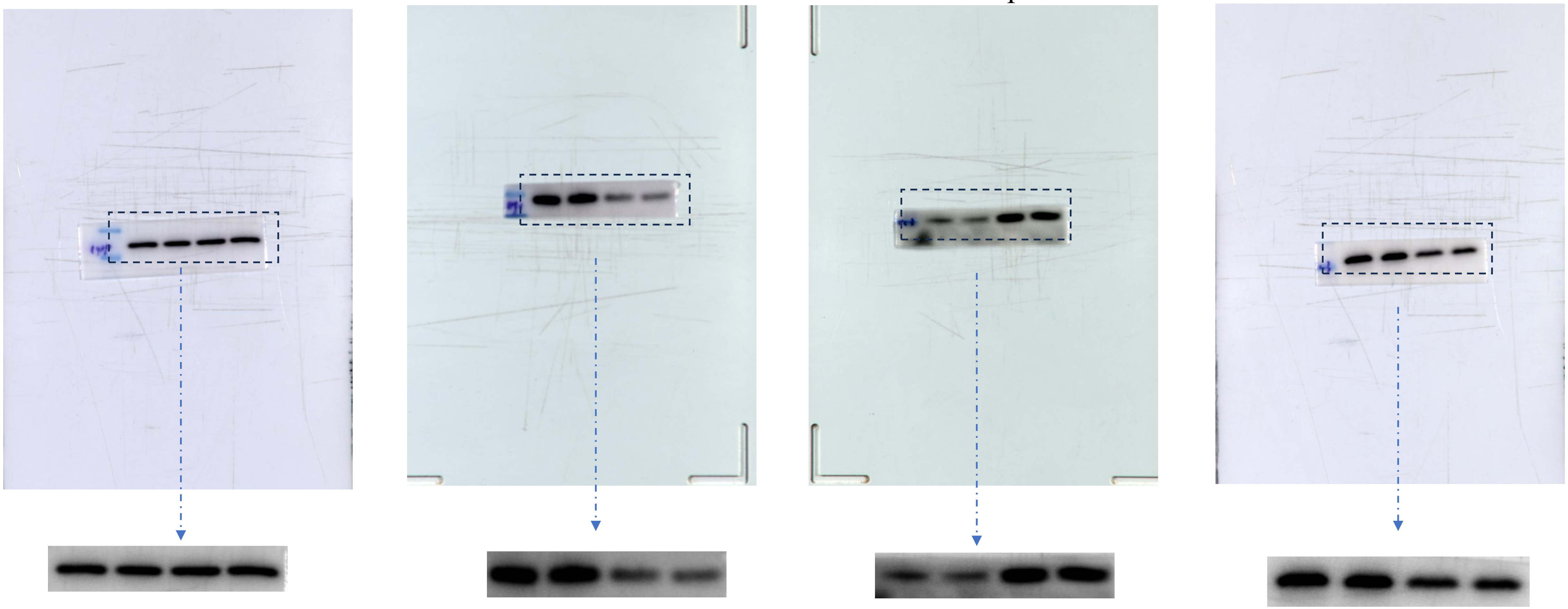

Fig 1B +s2D Repeat 2

Marker Ctrl Ctrl CVB3 CVB3

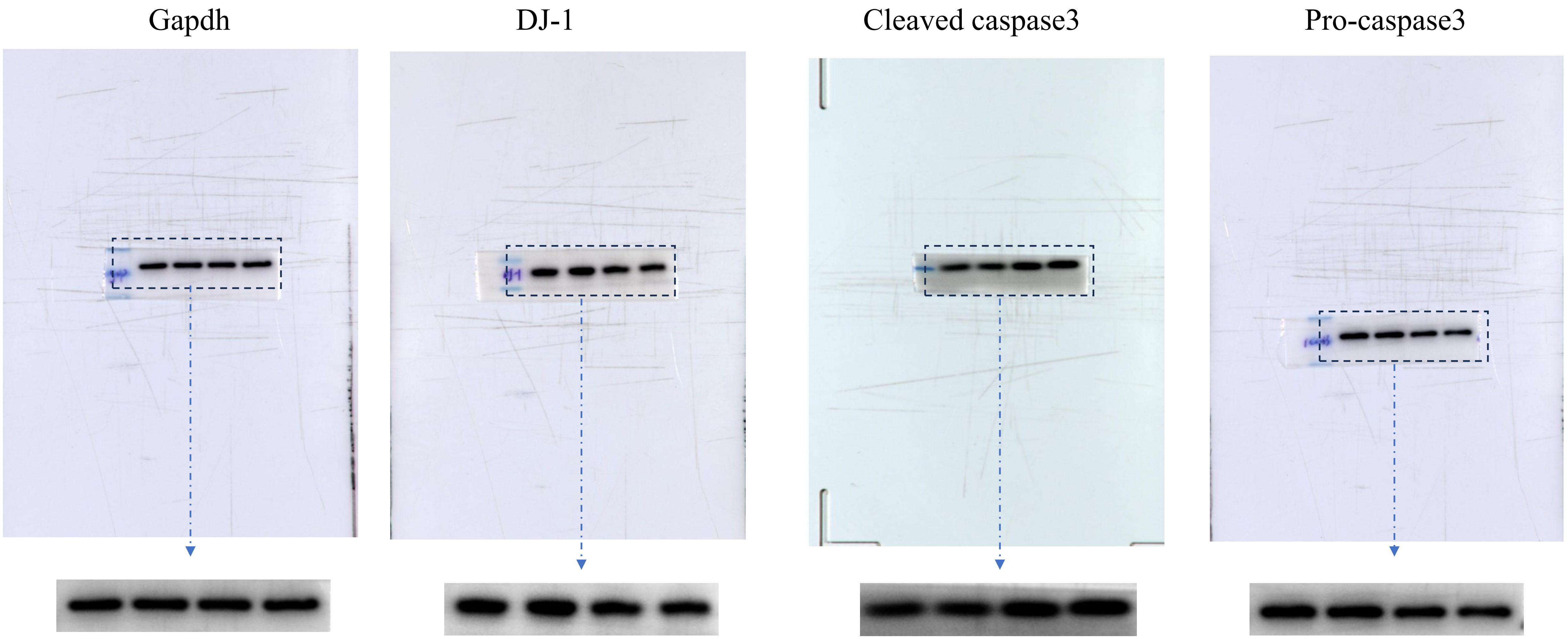

Fig 1B +s2D Repeat 3

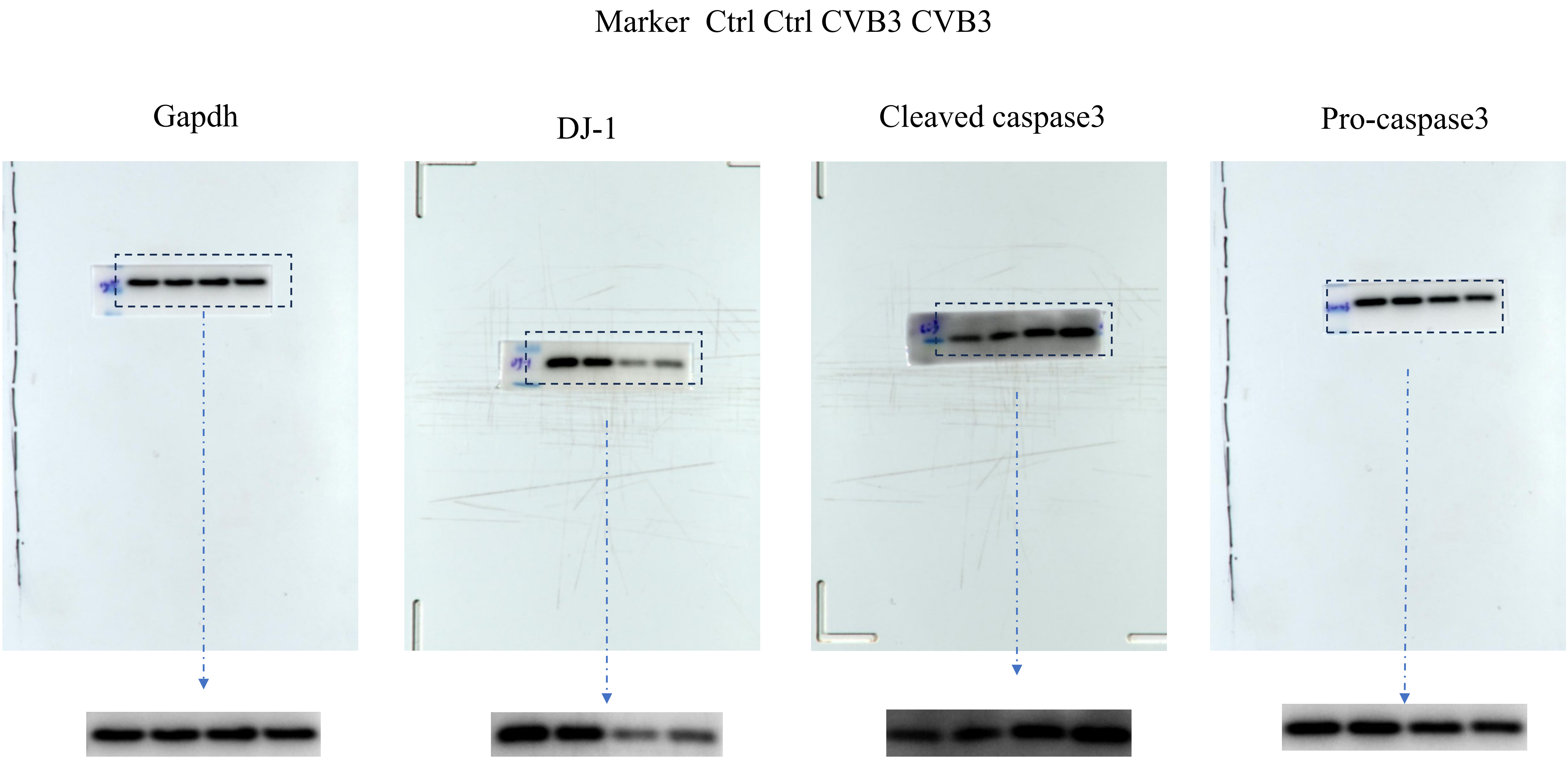

Fig 1D+s1B Repeat 1

Marker Ctrl Ctrl VMC VMC

Gapdh   DJ-1   Cleaved caspase3   Pro-caspase3

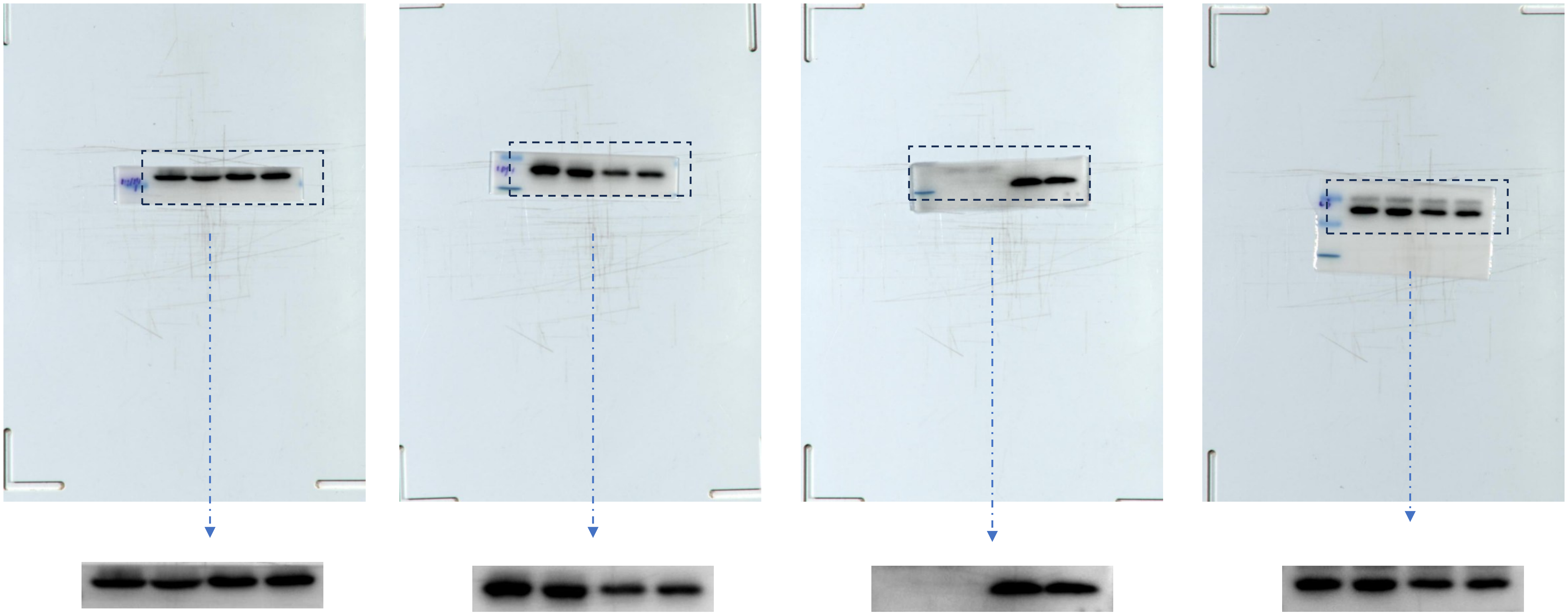

Fig 1D +s1B Repeat 2

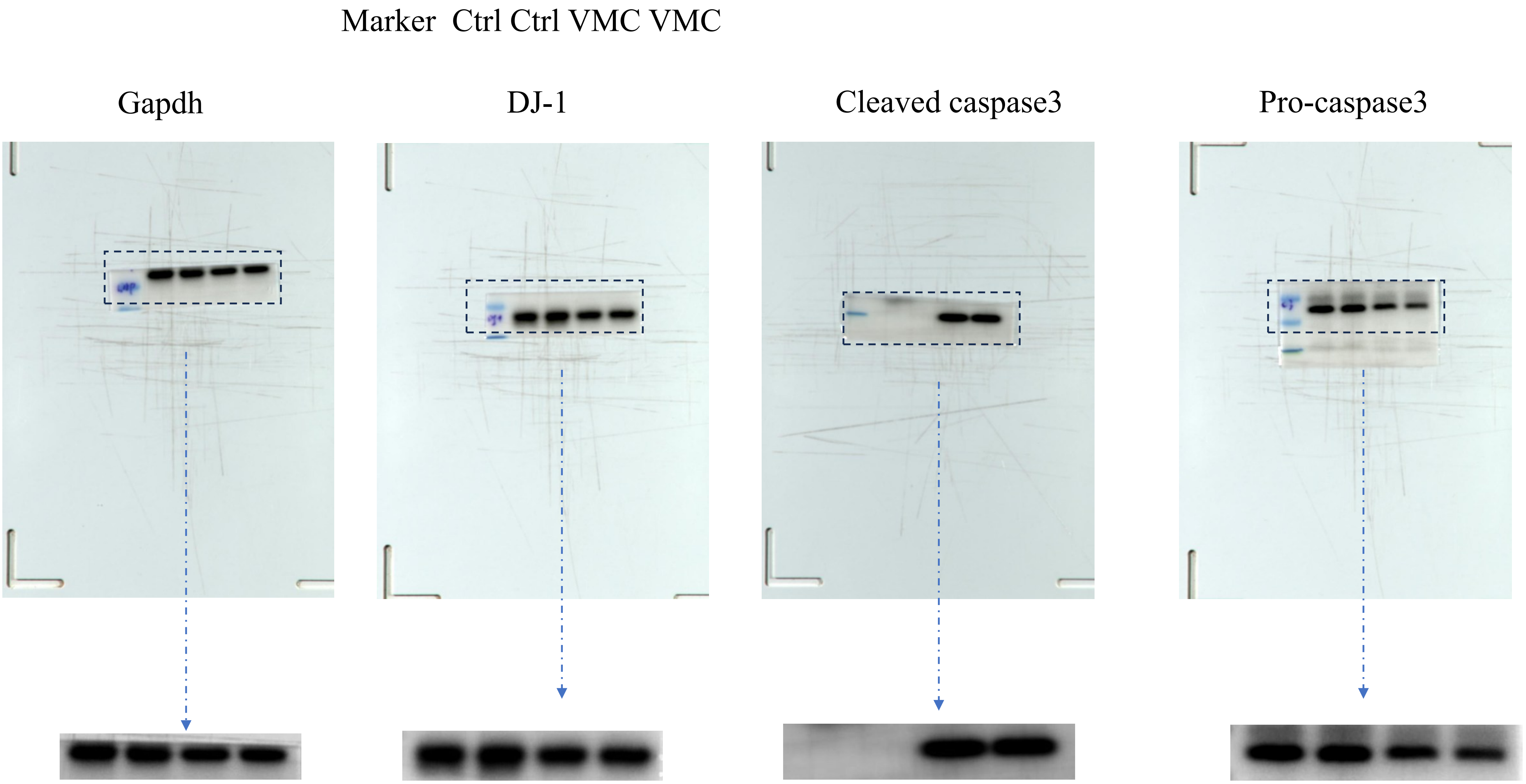

Fig 1D +s1B Repeat 3   Marker Ctrl Ctrl VMC VMC

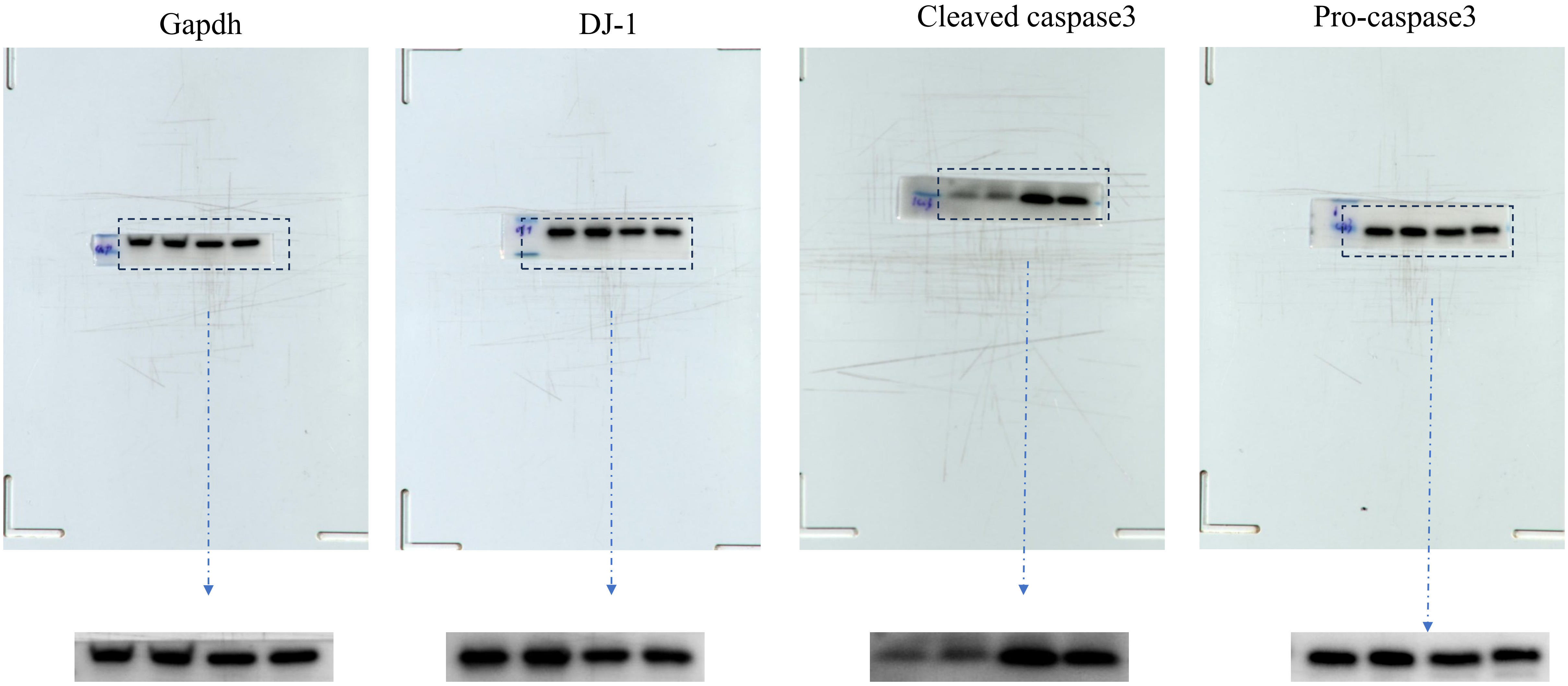

Fig 3C Repeat 1

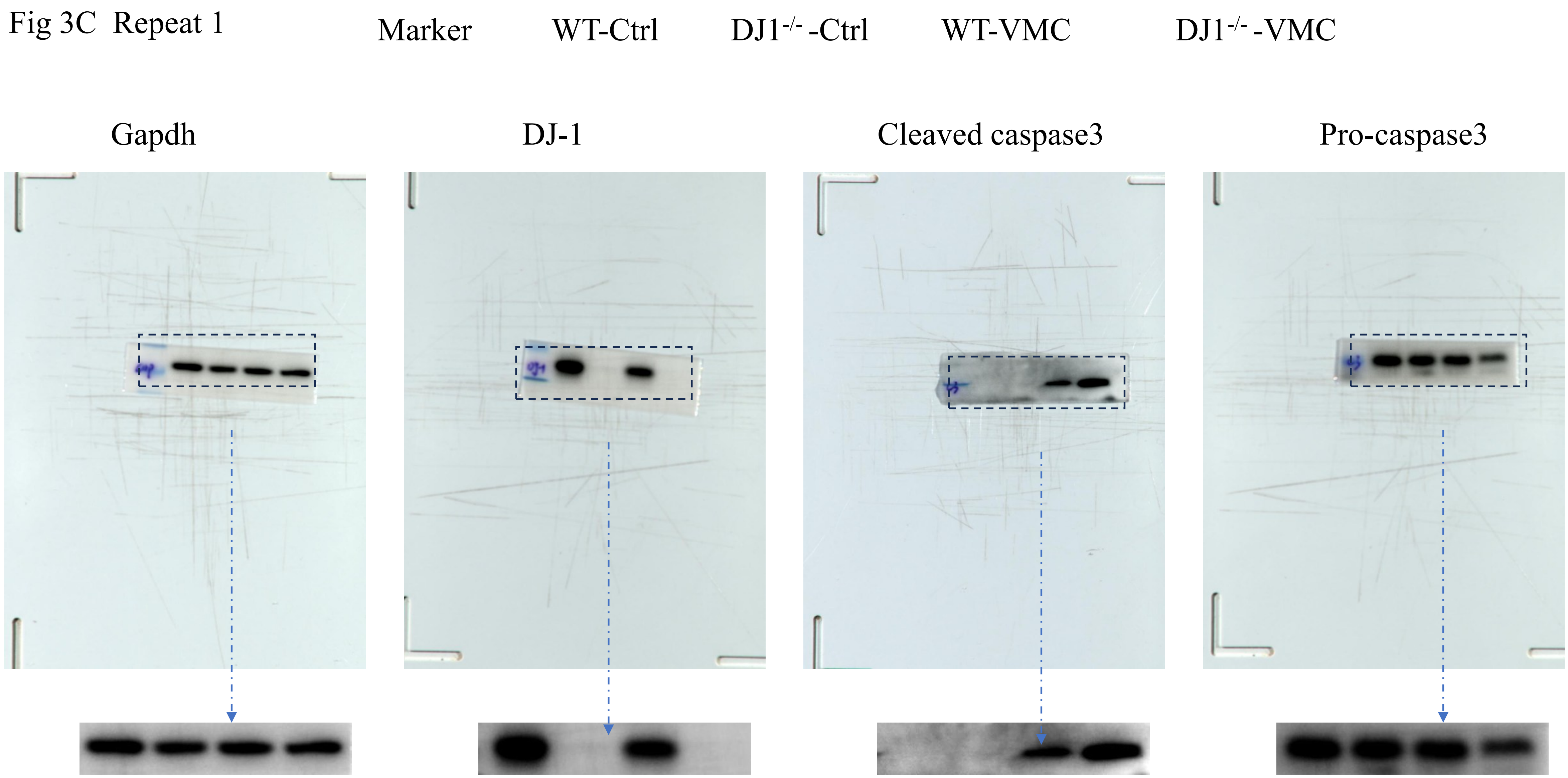

Fig 3C Repeat 2

Marker WT-Ctrl DJ1$^{-/-}$ -Ctrl WT-VMC DJ1$^{-/-}$ -VMC

Gapdh DJ-1 Cleaved caspase3 Pro-caspase3

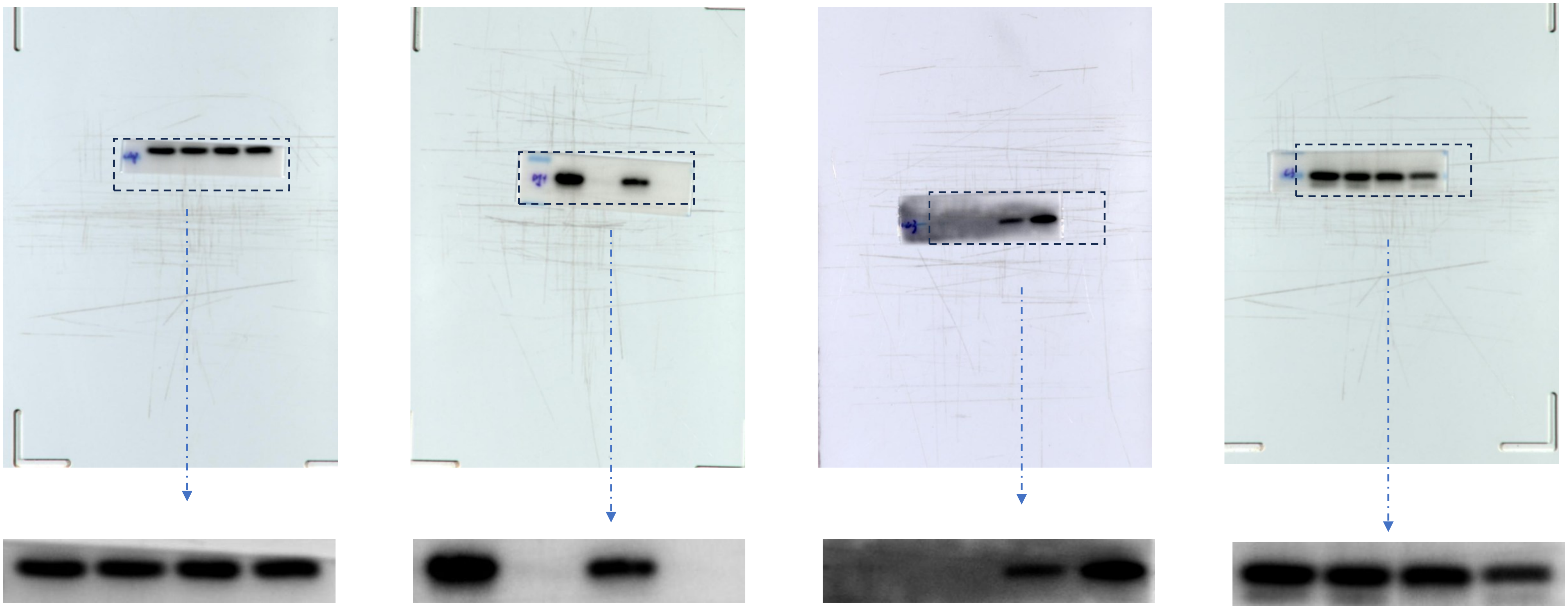

Fig 3C Repeat 3

Marker WT-Ctrl DJ1$^{-/-}$-Ctrl WT-VMC DJ1$^{-/-}$-VMC

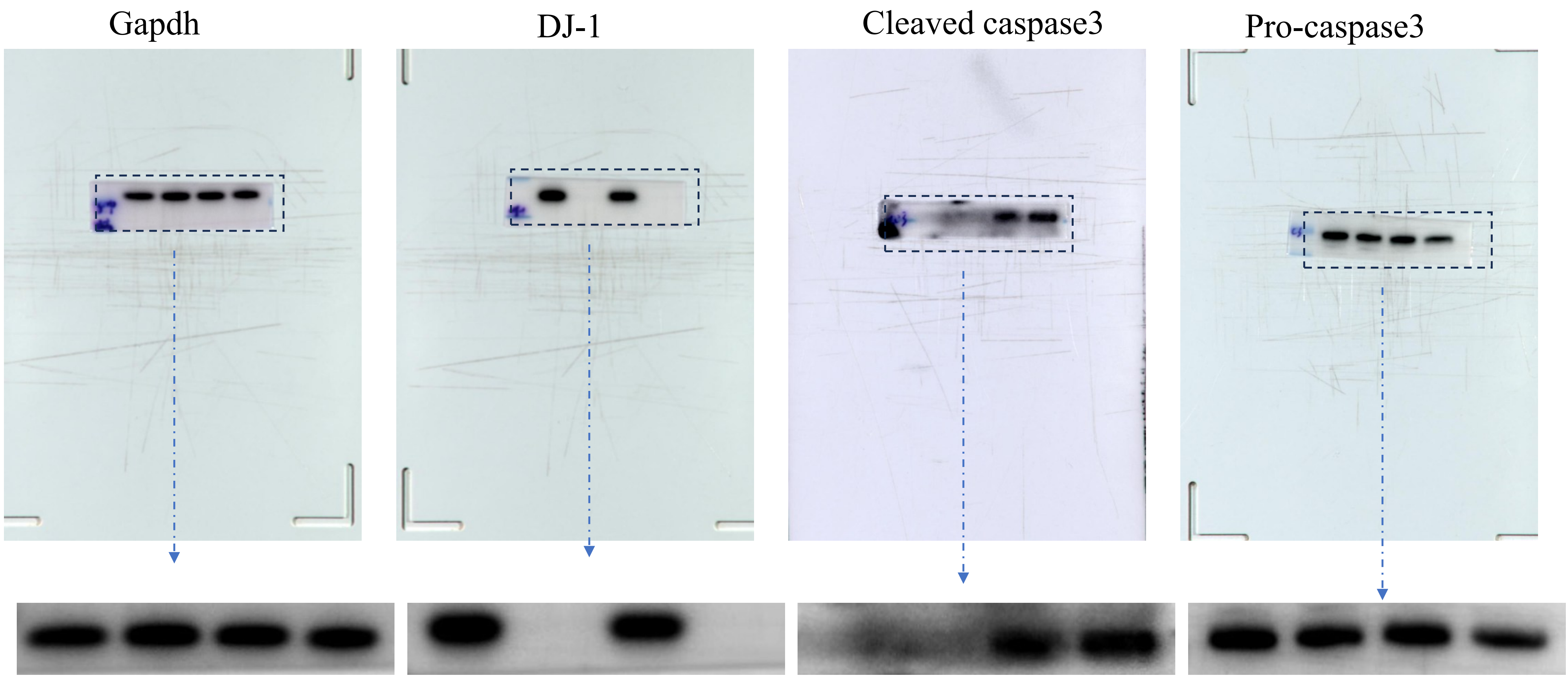

Fig 3E Repeat 1

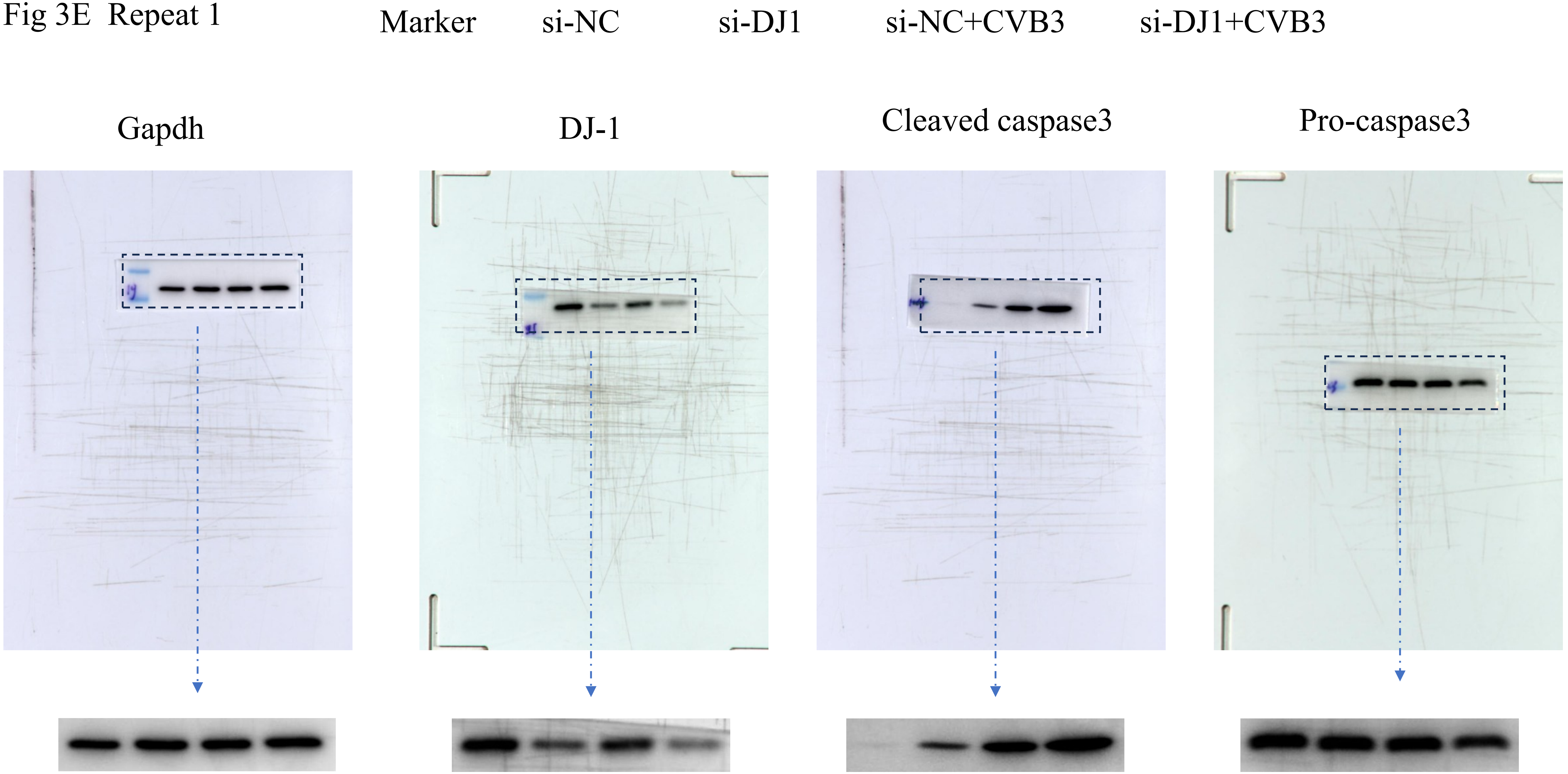

Fig 3E Repeat 2

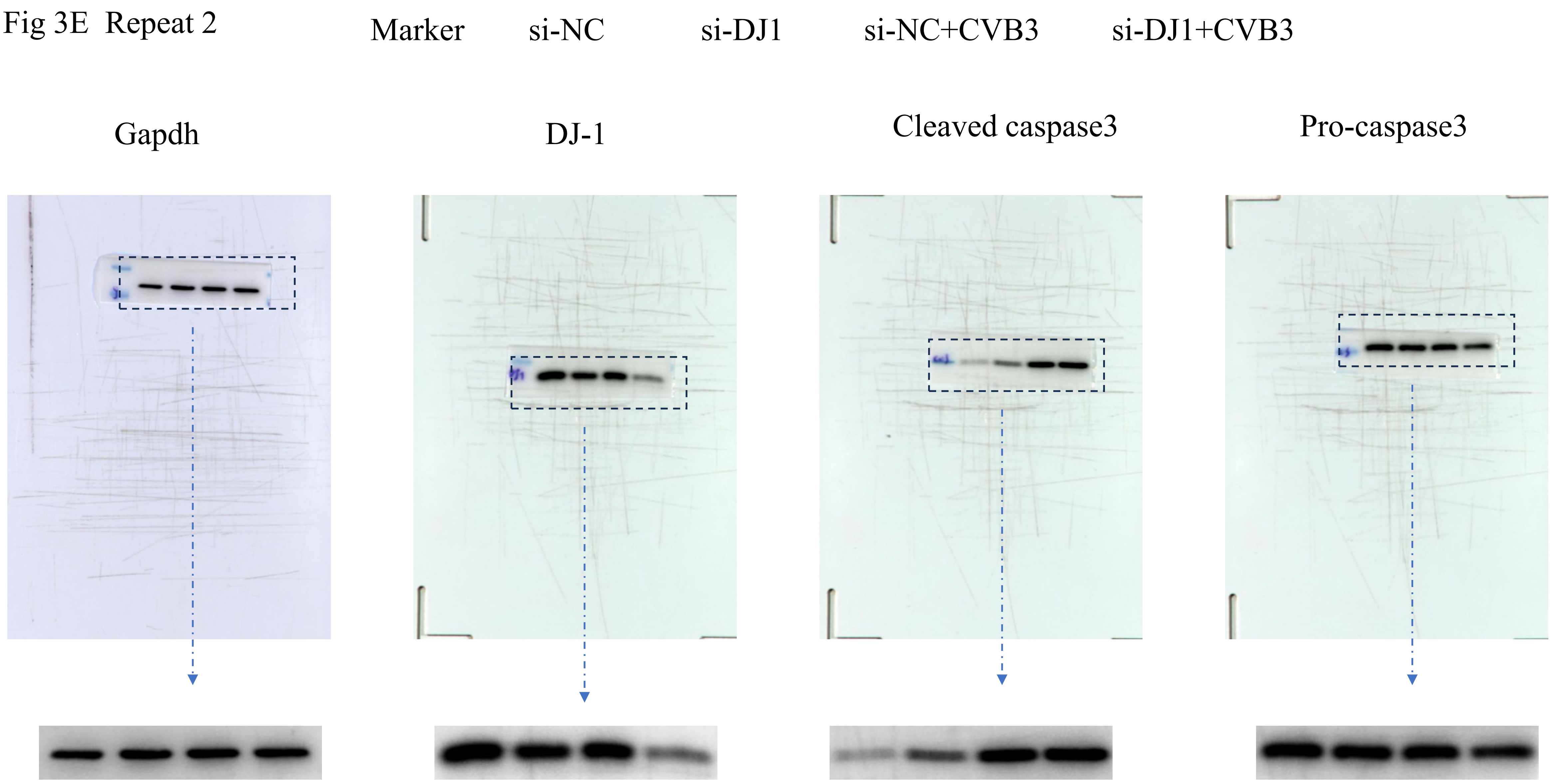

Fig 3E Repeat 3

Marker si-NC si-DJ1 si-NC+CVB3 si-DJ1+CVB3

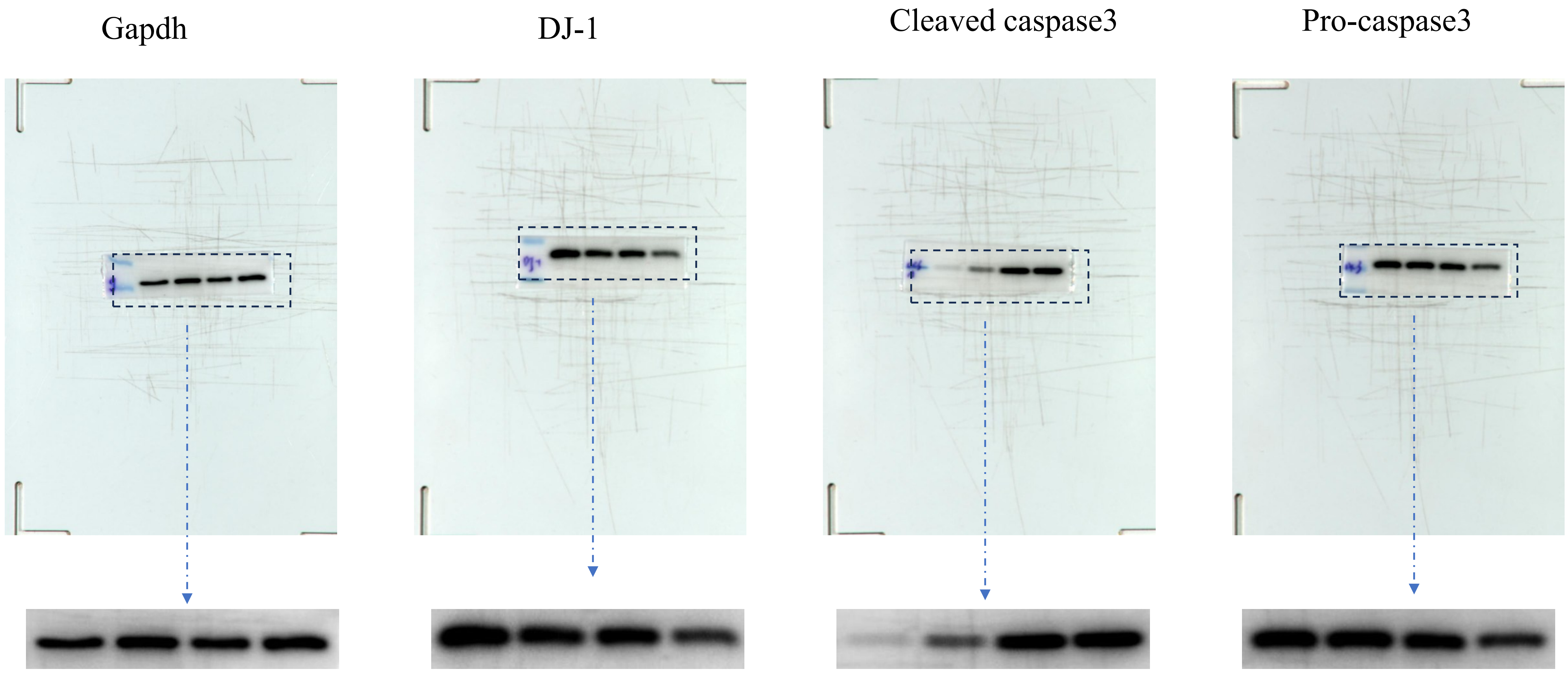

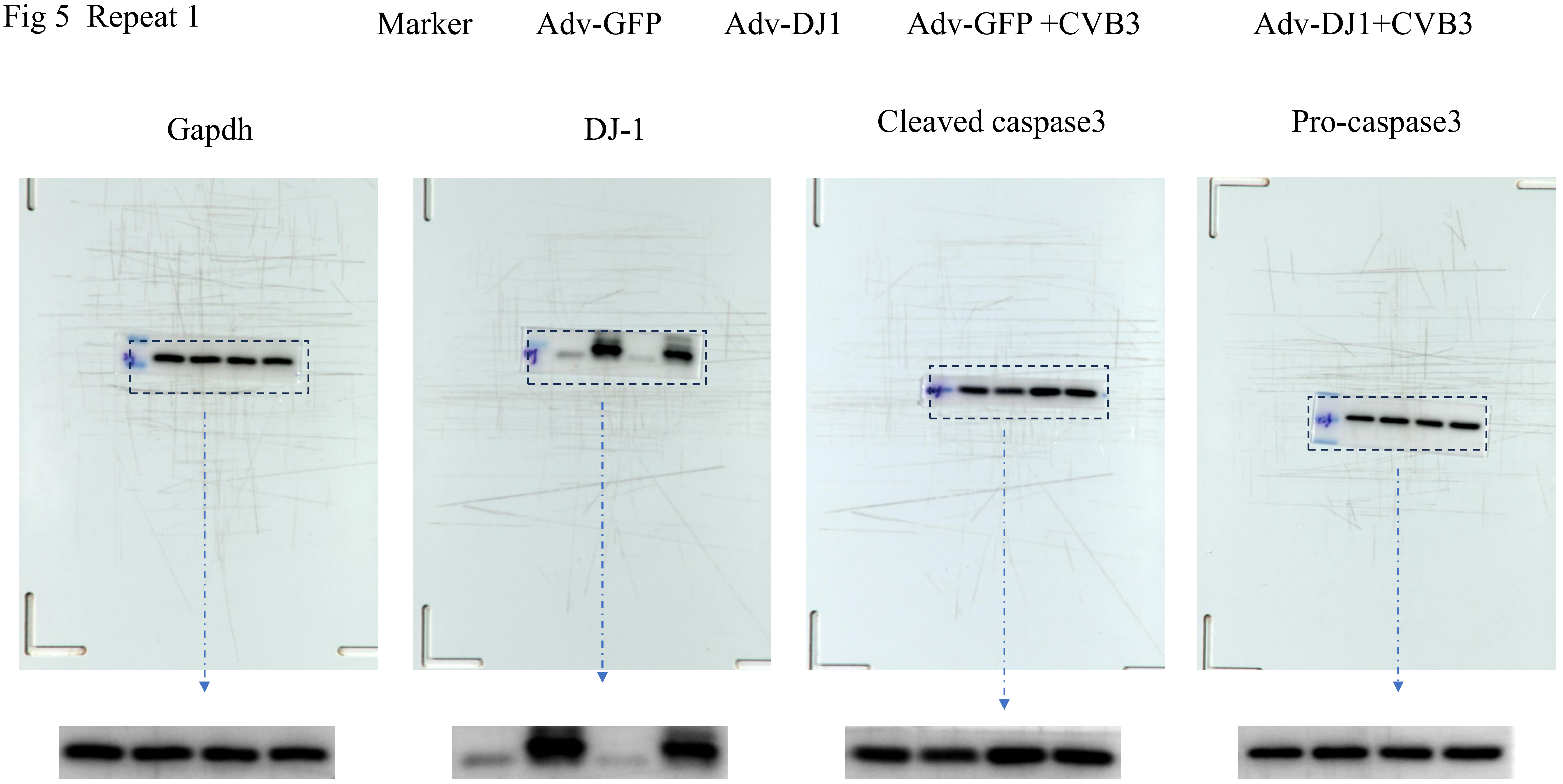

Fig 5 Repeat 1

Fig 5 Repeat 2

Marker Adv-GFP Adv-DJ1 Adv-GFP +CVB3 Adv-DJ1+CVB3

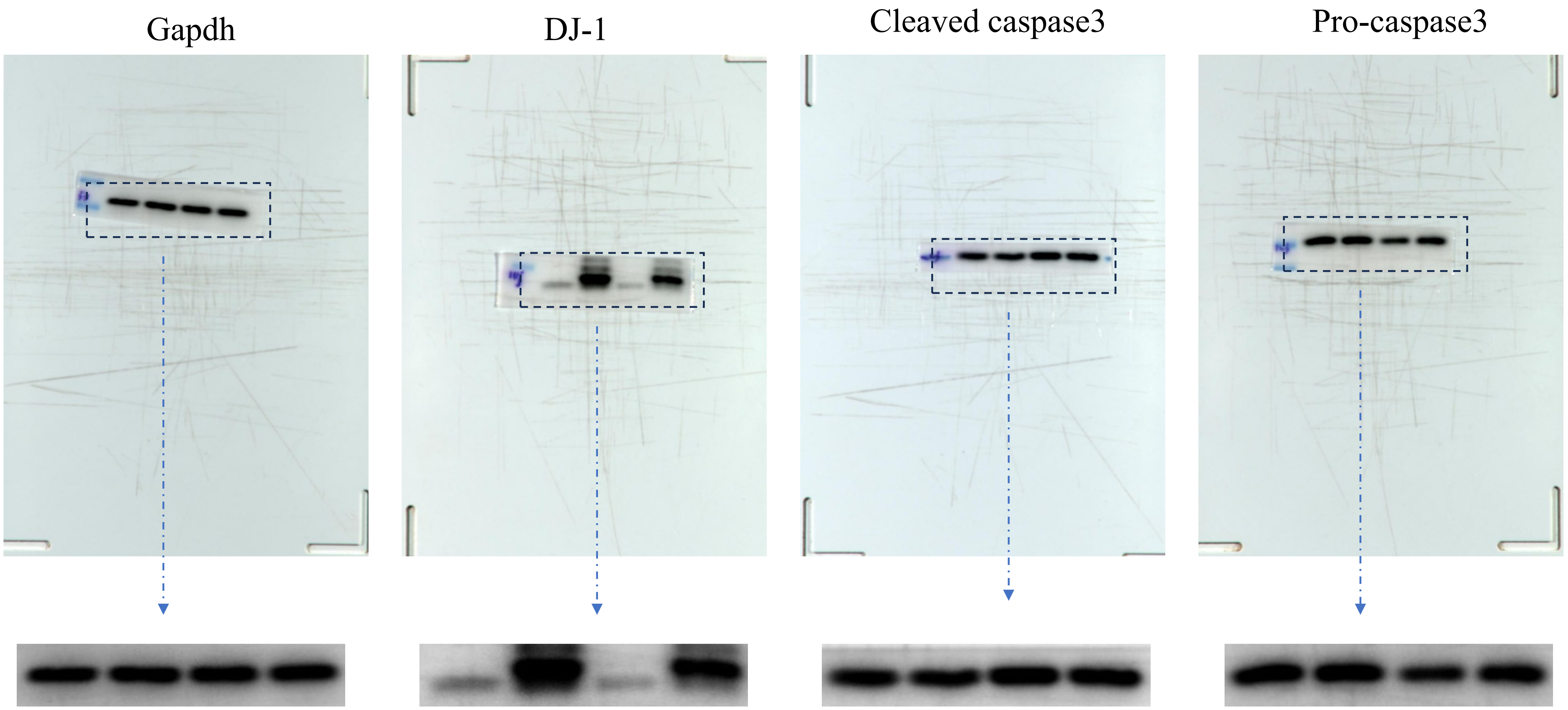

Fig 5 Repeat 3

Marker Adv-GFP Adv-DJ1 Adv-GFP +CVB3 Adv-DJ1+CVB3

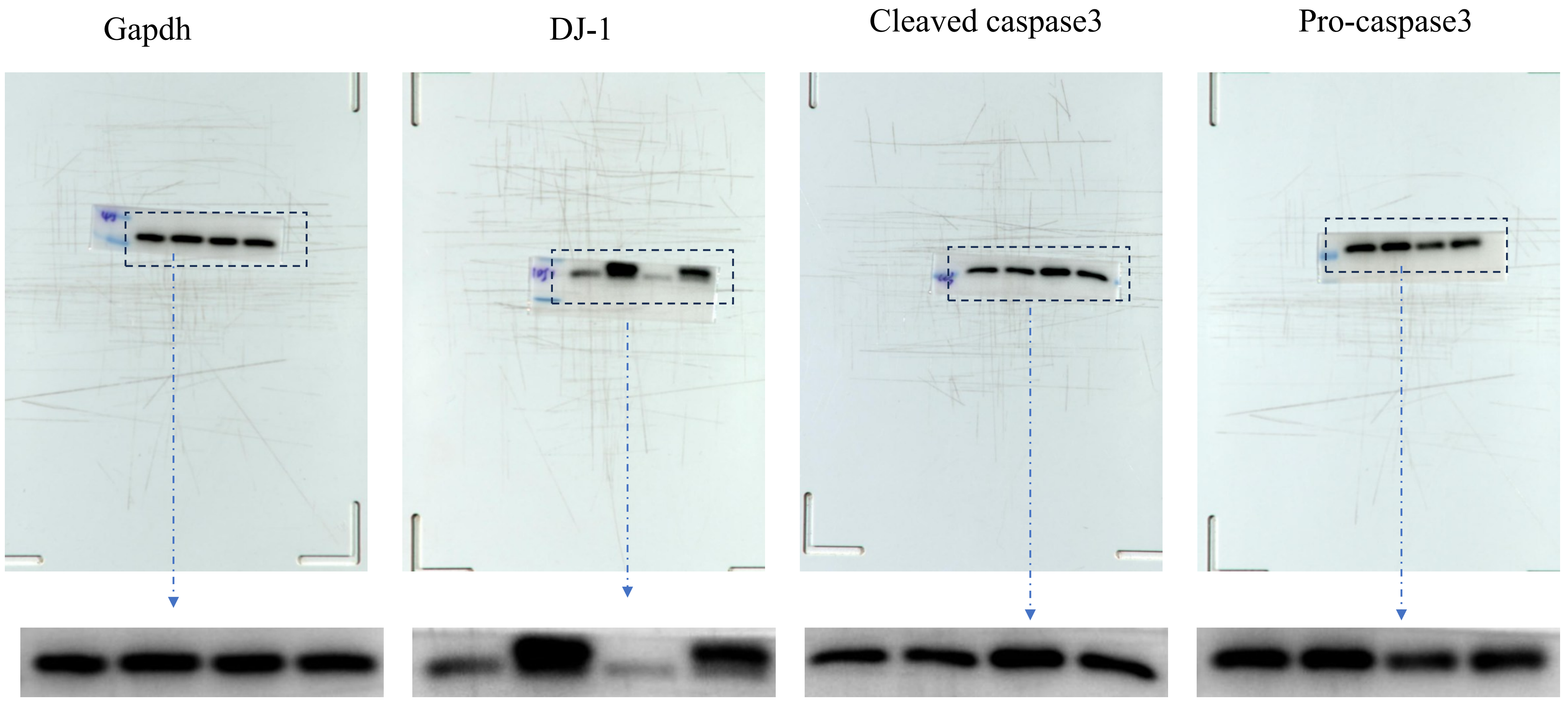

Fig 6E Repeat 1

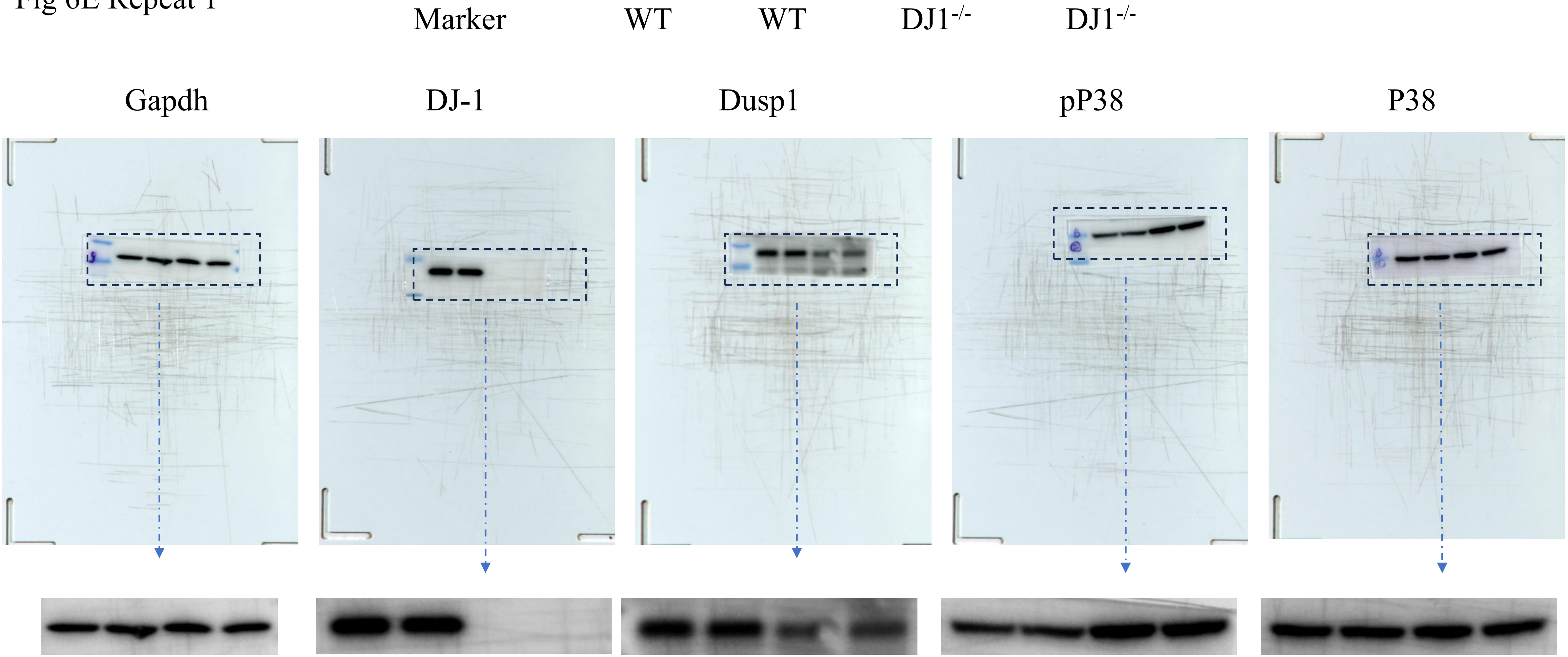

Fig 6E Repeat 2

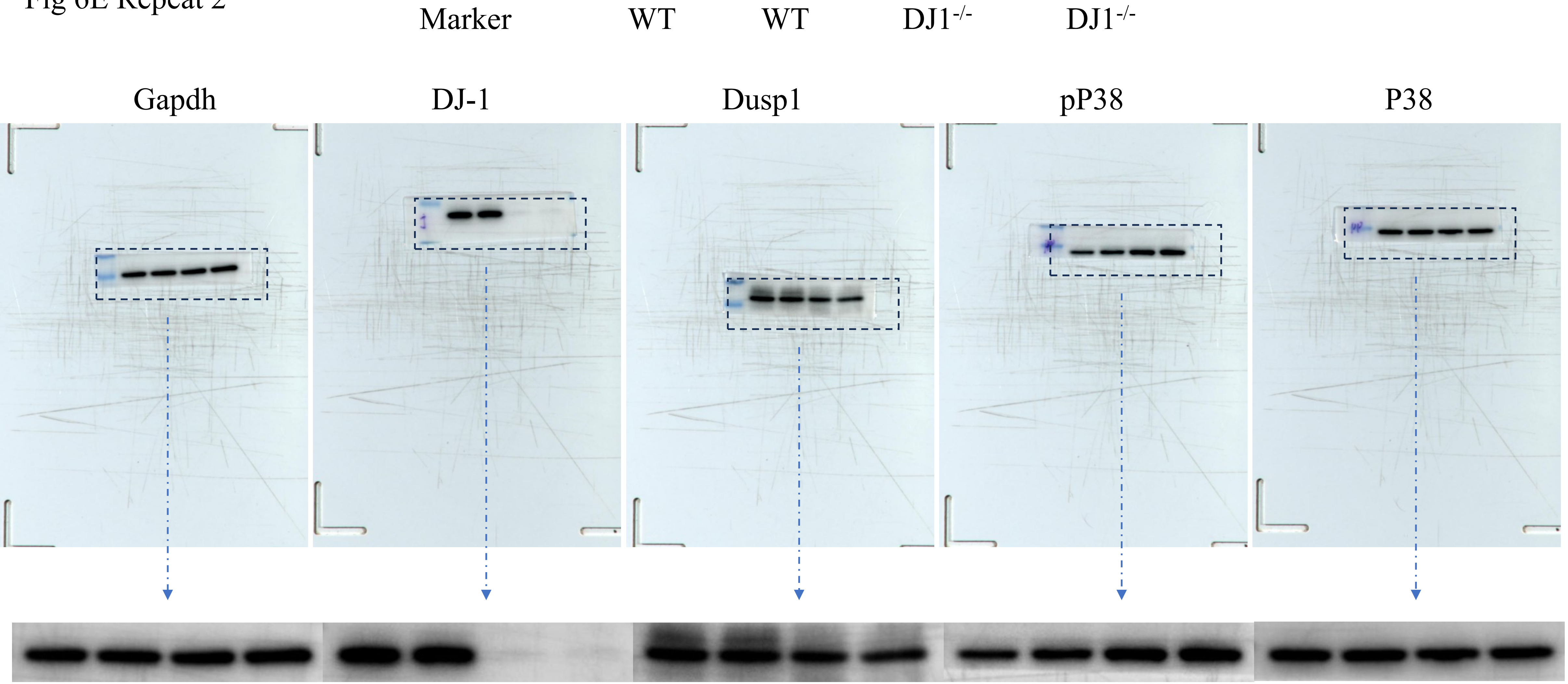

Fig 6E Repeat 3

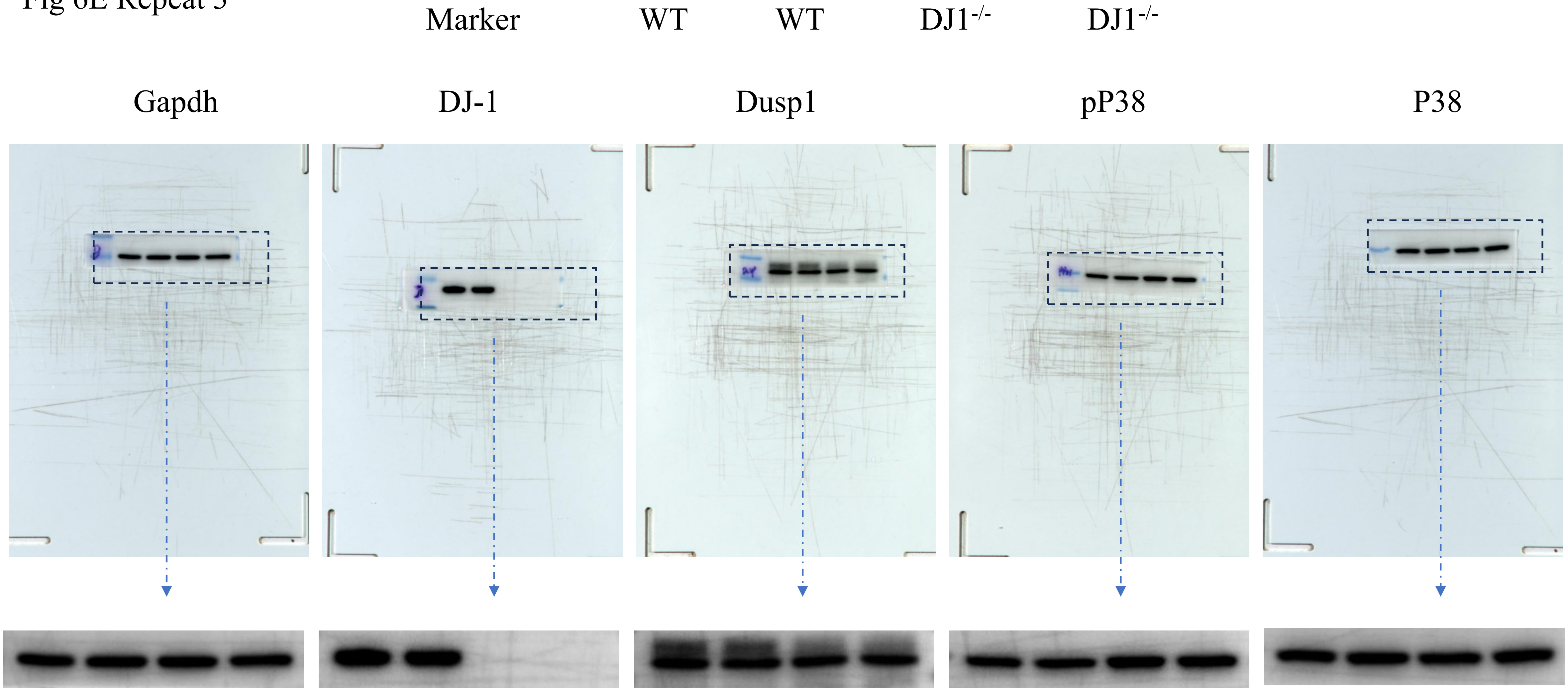

Fig 6G Repeat 1

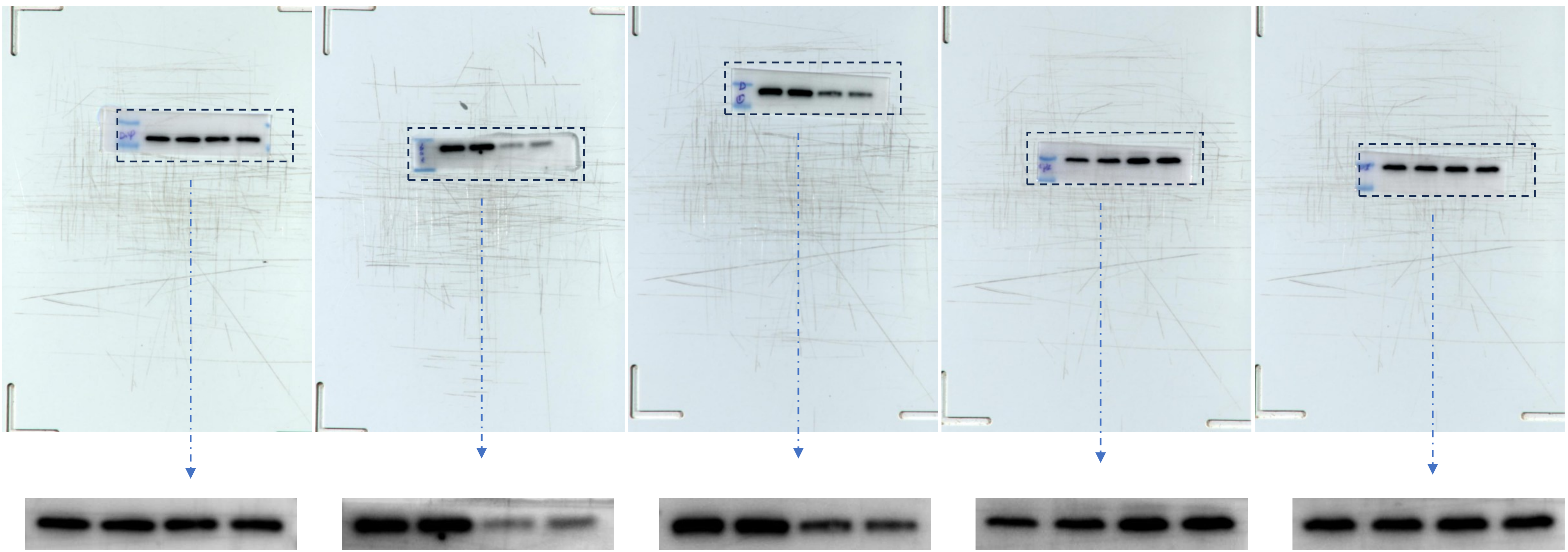

Fig 6G Repeat 2

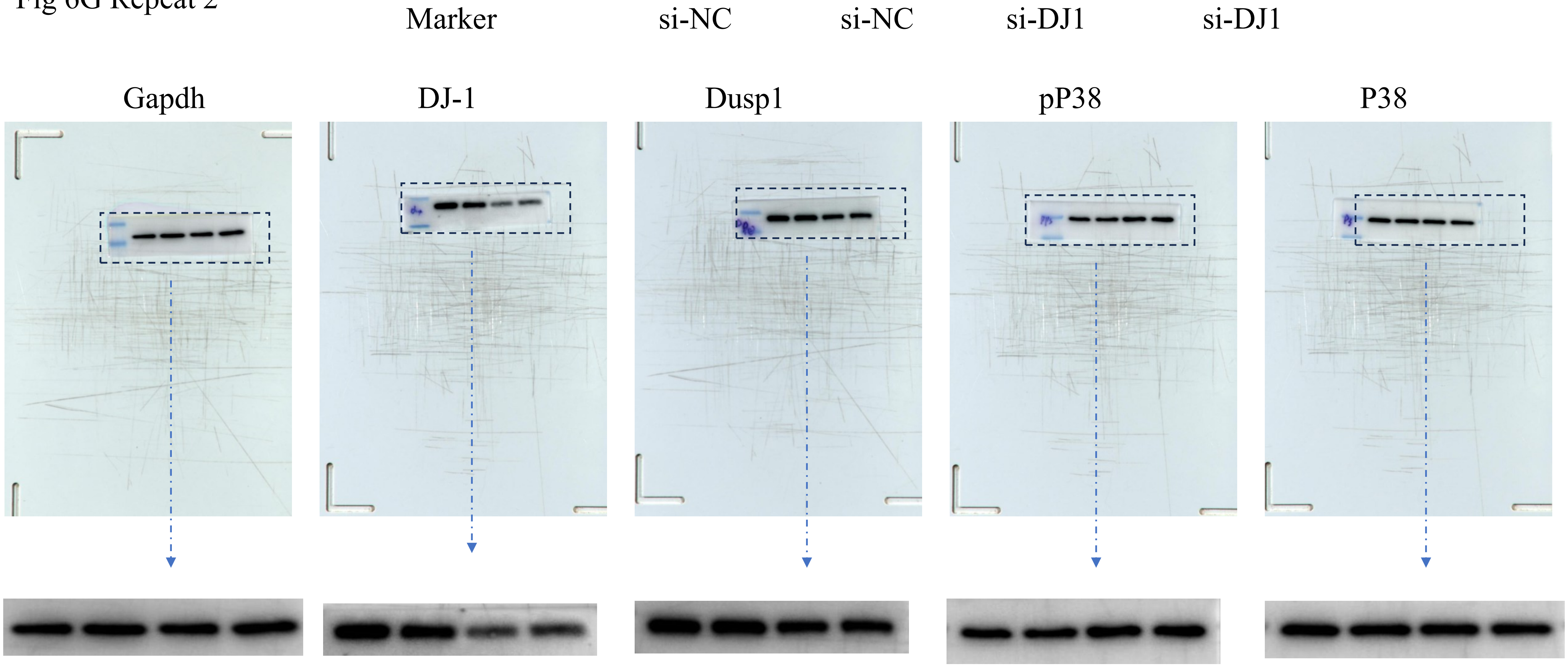

Fig 6G Repeat 3

Marker si-NC si-NC si-DJ1 si-DJ1

Gapdh DJ-1 Dusp1 pP38 P38

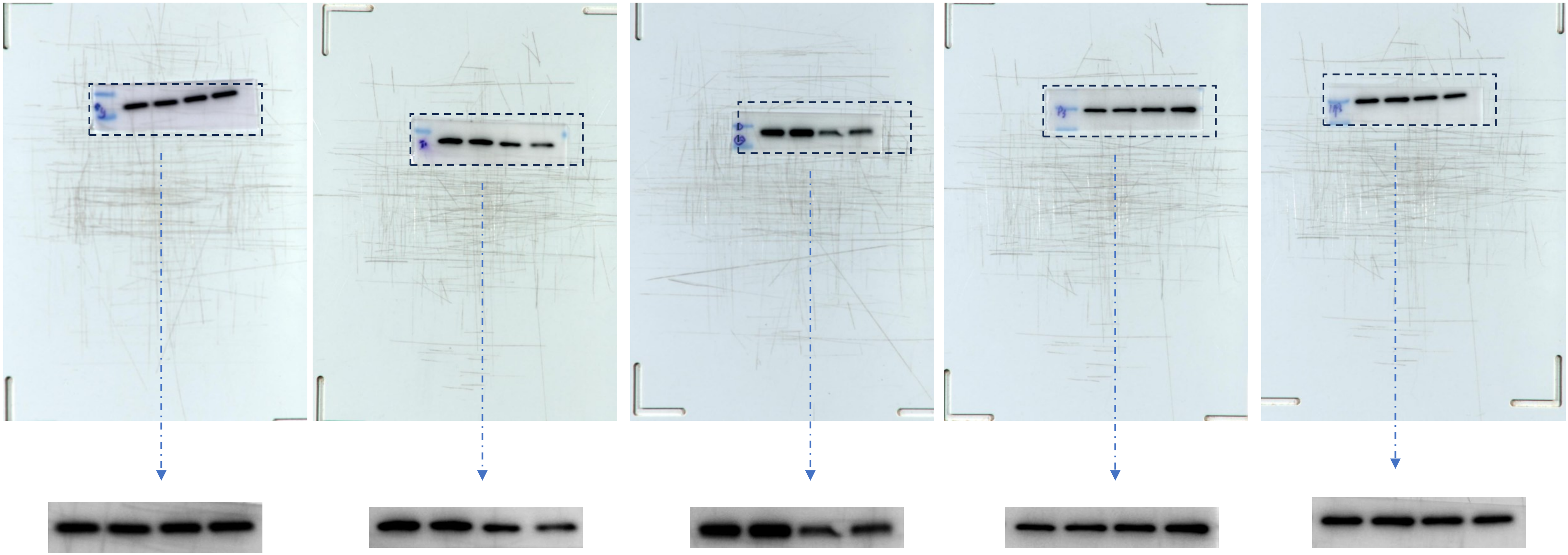

Fig 7A Repeat 1

Marker WT-Ctrl DJ1$^{-/-}$-Ctrl WT-VMC DJ1$^{-/-}$-VMC

Gapdh DJ-1 Dusp1

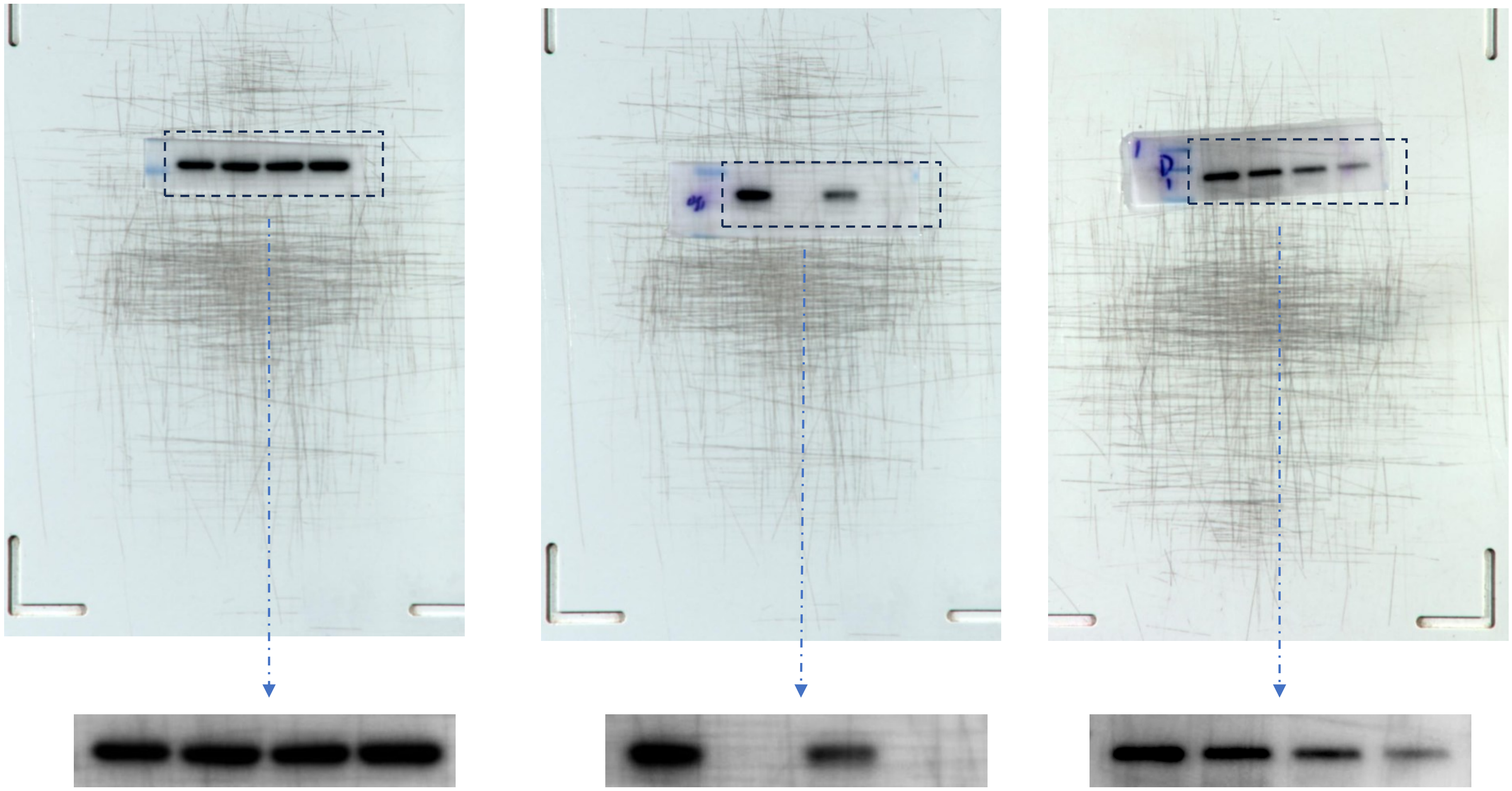

Fig 7A Repeat 2

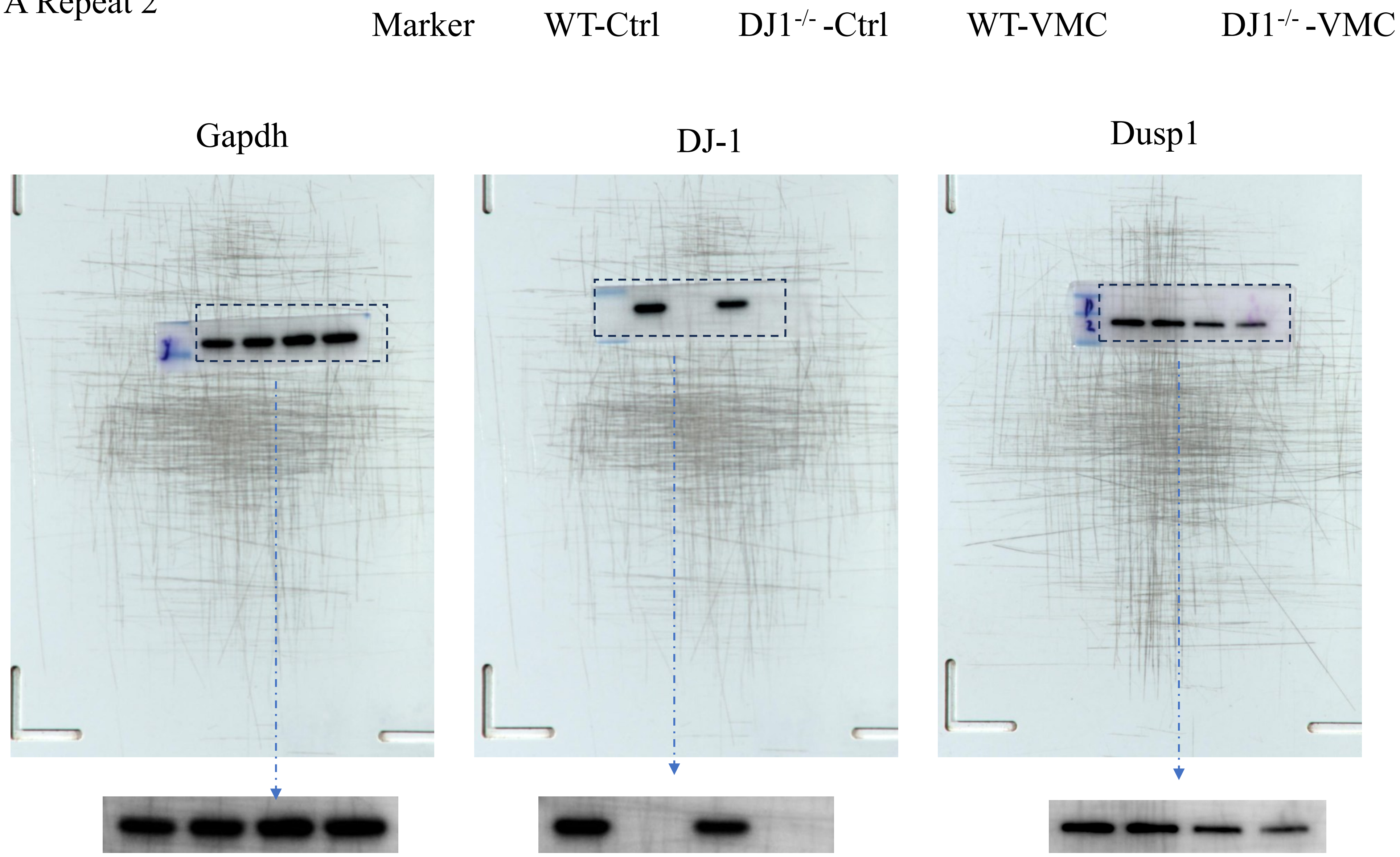

Fig 7A Repeat 3

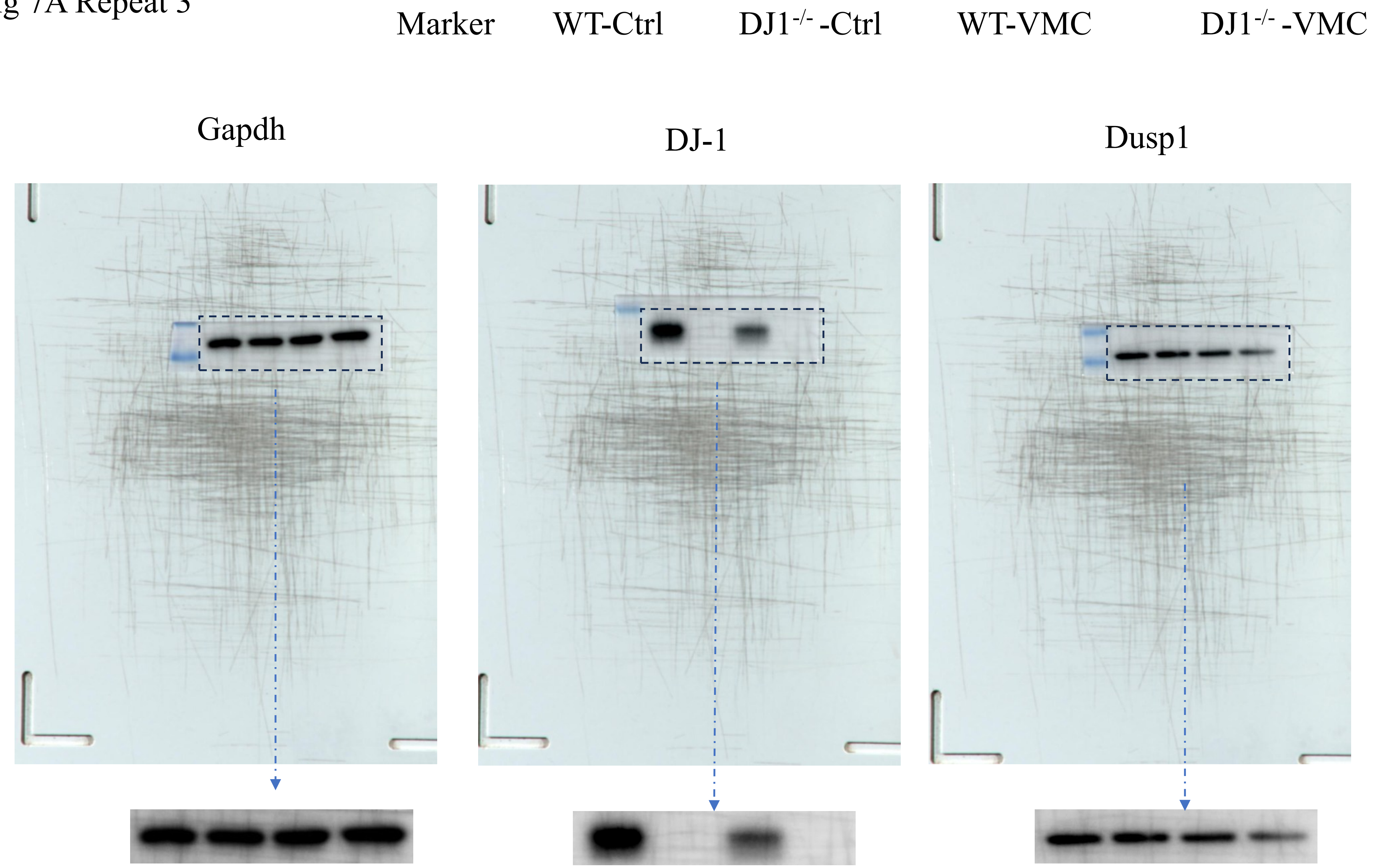

Fig 7E Repeat 1

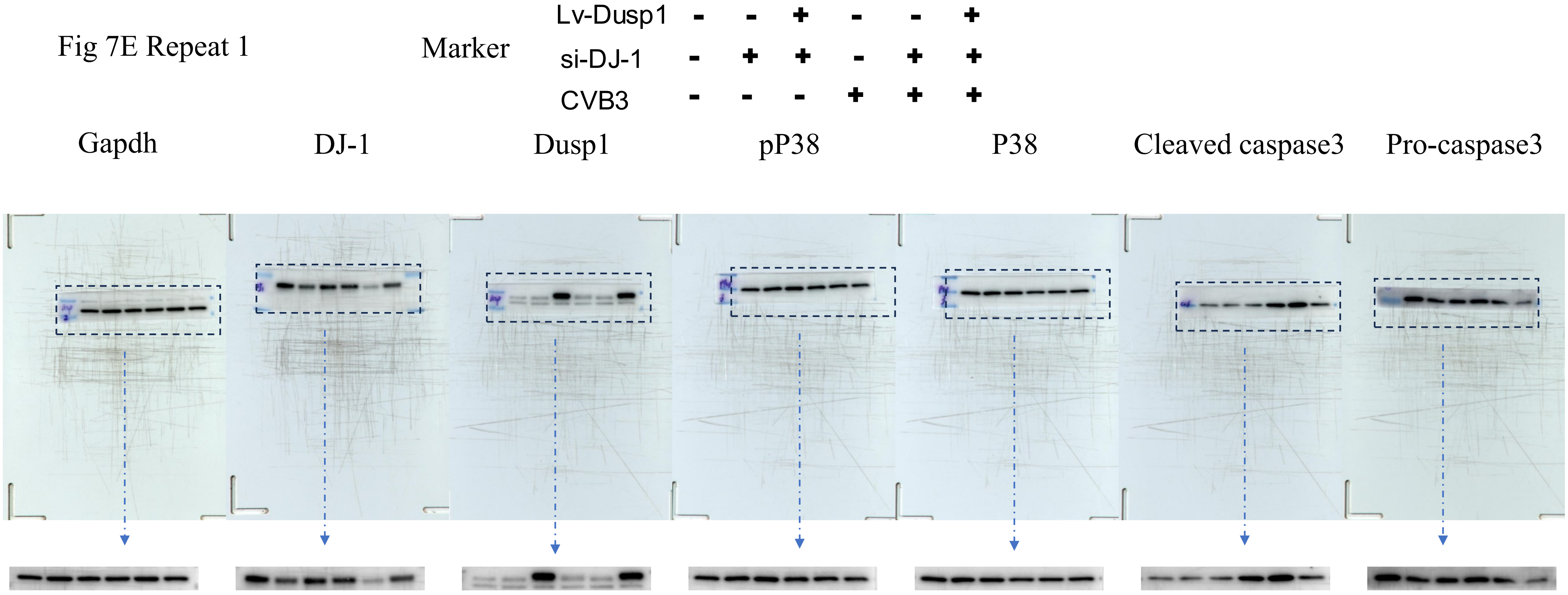

Fig 7E Repeat 2

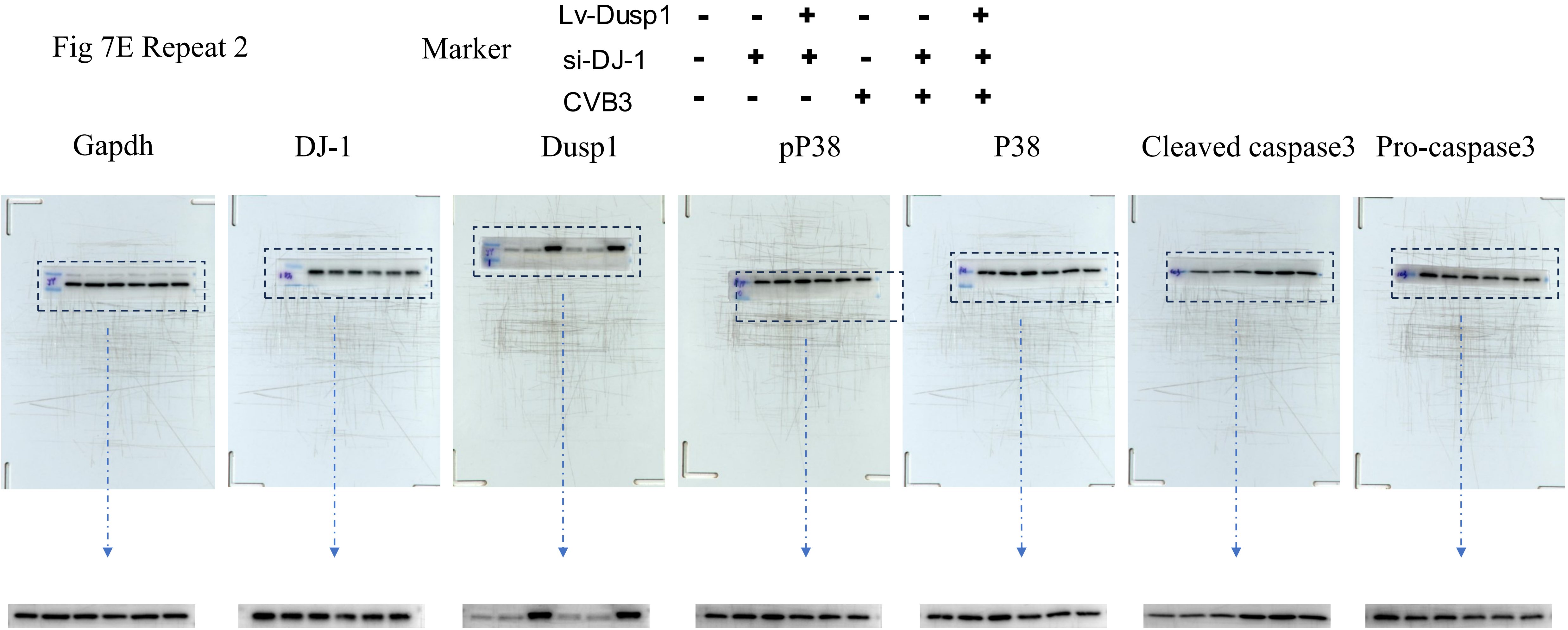

Fig 7E Repeat 3

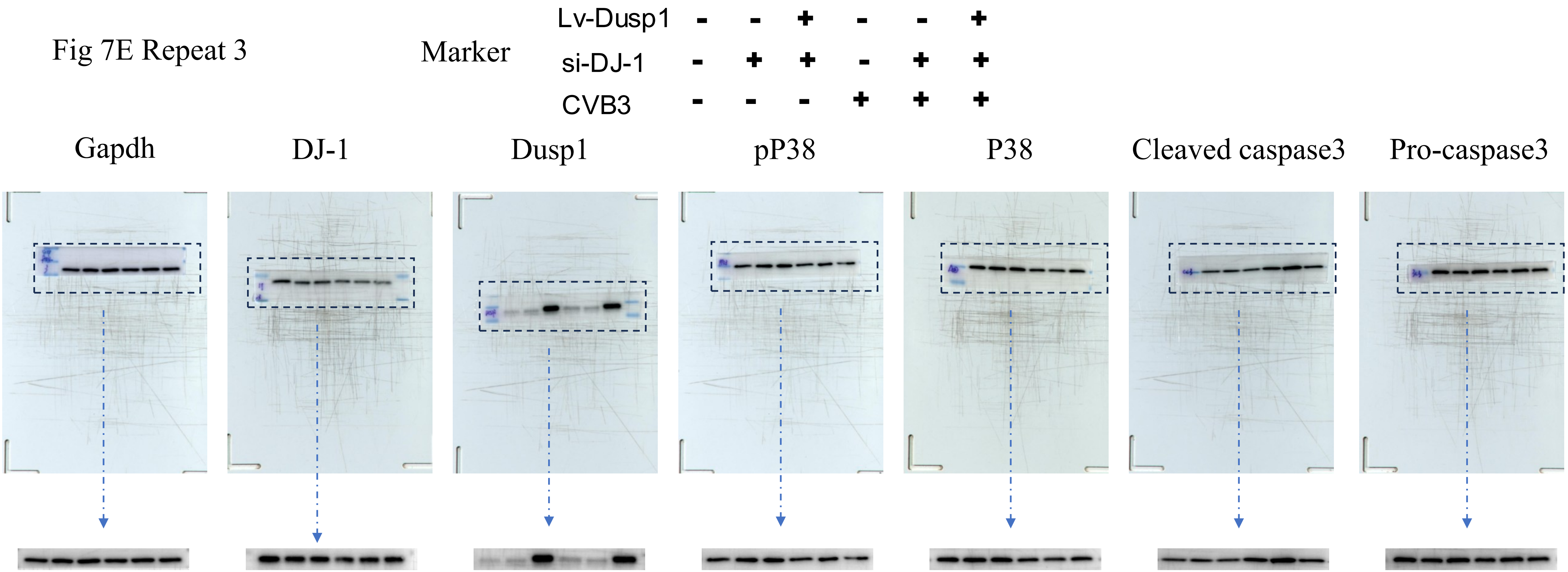

Fig s1 Repeat 1

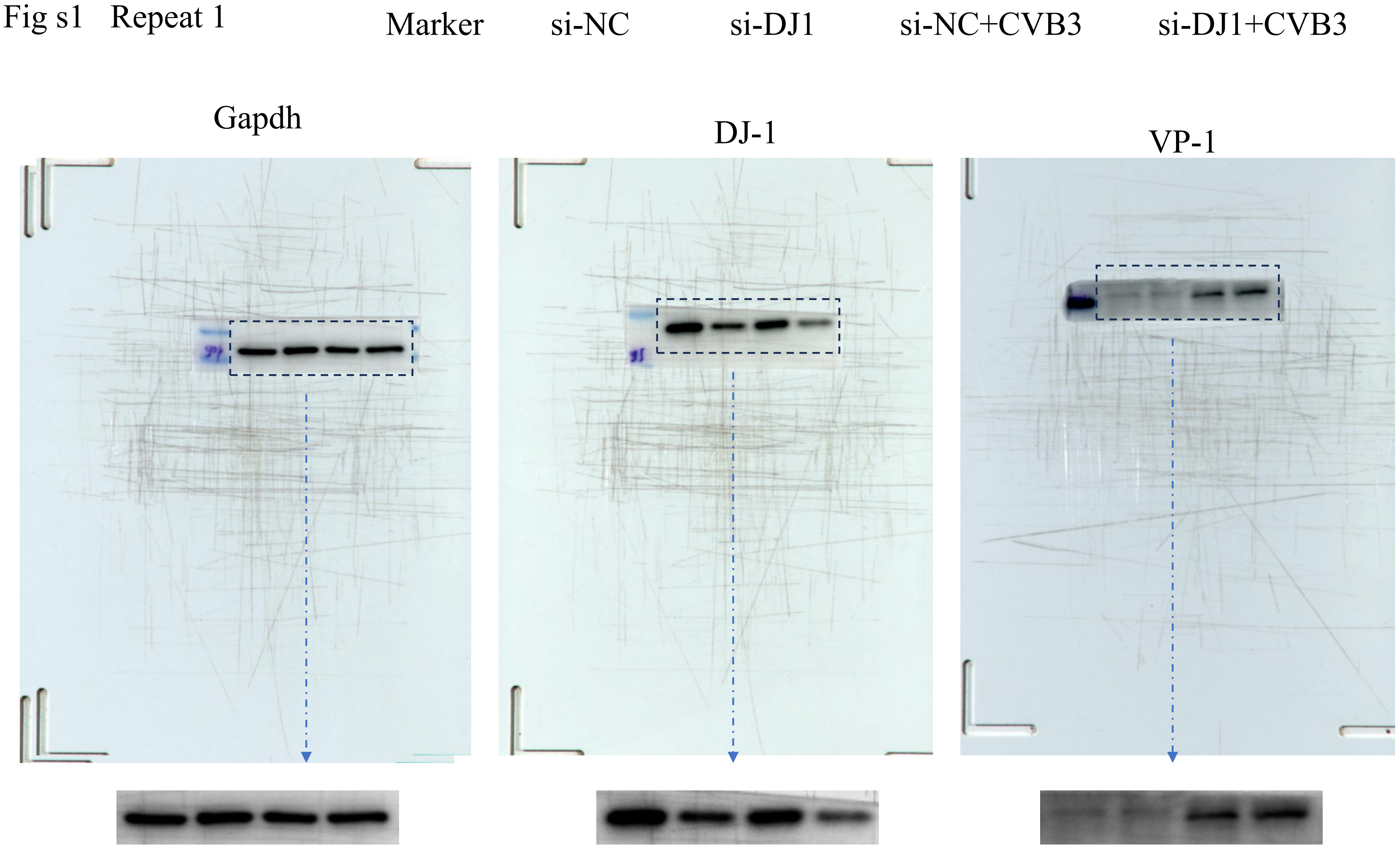

Fig s1 Repeat 2 Marker si-NC si-DJ1 si-NC+CVB3 si-DJ1+CVB3

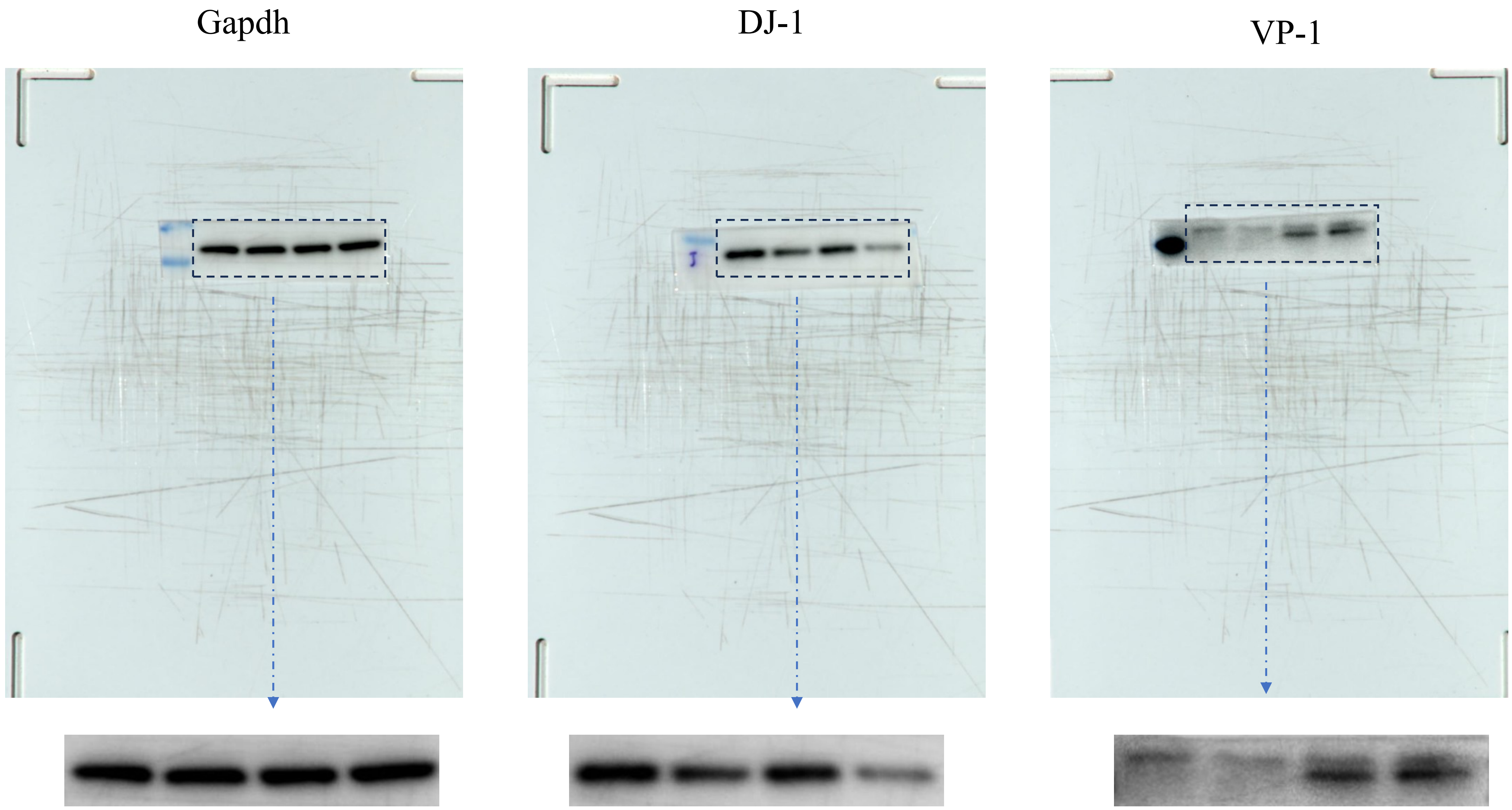

Fig s1 Repeat 3

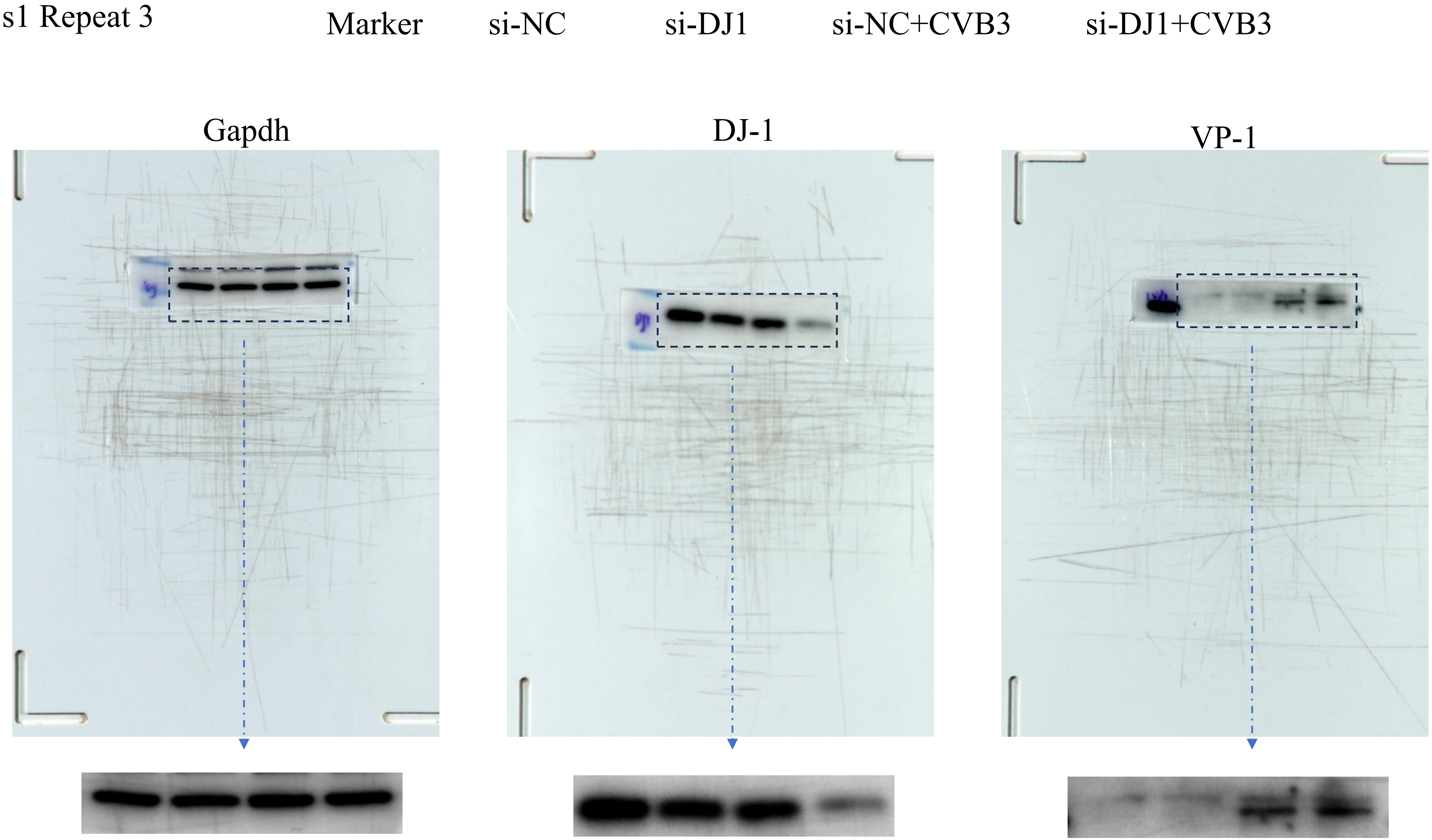

Supplement: Supplementary file 8 — Full and uncropped western blots [file 41419_2025_8185_MOESM8_ESM.pdf]
